# Machine learning prediction of postoperative pulmonary embolism: a multicenter external validation study highlighting inflammatory response and intraoperative hemodynamics

**DOI:** 10.3389/fcvm.2026.1870642

**Published:** 2026-06-18

**Authors:** Shunpeng He, Yuan Liu, Yilin Wu

**Affiliations:** 1 Department of Emergency Medicine, Sir Run Run Shaw Hospital, Zhejiang University School of Medicine, Hangzhou, China; 2 Department of General Surgery, The Affiliated Tengzhou Central People's Hospital of Xuzhou Medical University, Zao Zhuang, China

**Keywords:** inflammatory response, machine learning, multicenter study, predictive model, pulmonary embolism

## Abstract

**Background:**

Postoperative pulmonary embolism (PE) remains a rare but life-threatening complication after surgery. Early identification of high-risk patients is essential but remains challenging due to heterogeneous perioperative risk factors. This study aimed to develop and externally validate a machine learning-based prediction model for postoperative PE and to explore key clinical determinants using interpretable artificial intelligence.

**Methods:**

This multicenter retrospective study included surgical patients from six hospitals between January 2020 and January 2025. Patients were divided into internal and external datasets according to hospital source. A total of 3,494 patients were included, with 2005 in the internal cohort and 1,489 in the external validation cohort. Candidate variables were selected using a hybrid strategy combining univariate and multivariate logistic regression with feature importance ranking from five machine learning algorithms, including XGBoost, Random Forest, Support Vector Machine, K-Nearest Neighbors, and Multilayer Perceptron. Model performance was evaluated using receiver operating characteristic (ROC) curves, calibration curves, decision curve analysis (DCA), Kolmogorov–Smirnov (KS) statistics, and confusion matrices. K-fold cross-validation and external validation were further performed to assess model robustness. SHapley Additive exPlanations (SHAP) were used for model interpretability and individualized prediction.

**Results:**

A total of 48 patients (1.38%) developed postoperative PE. The final selected predictors included age, body mass index (BMI), malignancy history, prolonged bed rest, surgery duration, intraoperative tachycardia, C-reactive protein (CRP), neutrophil-to-lymphocyte ratio (NLR), and postoperative D-dimer. Among five machine learning models, XGBoost demonstrated the best overall performance and stability, achieving superior discrimination and calibration. In the internal validation, the model showed strong predictive performance, and in the external validation cohort, it achieved an AUC of 0.925 (95% CI 0.877–0.972), with good calibration and favorable clinical net benefit on DCA. K-fold cross-validation confirmed model robustness with stable performance across resampling sets. SHAP analysis identified surgery duration, CRP level, malignancy history, age, BMI, postoperative D-dimer, NLR, and intraoperative tachycardia as the most influential predictors. Individual-level SHAP interpretation further demonstrated clinically meaningful risk attribution patterns for postoperative PE.

**Conclusion:**

We developed and externally validated a robust machine learning model for predicting postoperative pulmonary embolism across multiple surgical populations. The model demonstrated strong discrimination, good calibration, and favorable clinical utility. Importantly, SHAP-based interpretation revealed key perioperative inflammatory, thrombotic, and hemodynamic factors associated with PE risk, providing both predictive and mechanistic insights to support clinical decision-making.

## Introduction

Venous thromboembolism (VTE) encompasses a continuous pathophysiological spectrum comprising deep vein thrombosis (DVT) and pulmonary embolism (PE). DVT typically originates in the valvular sinuses of the deep veins of the lower extremities or within the venous plexus of the gastrocnemius muscle, where fibrin–platelet thrombi progressively propagate under the synergistic influence of altered hemodynamic shear stress, endothelial injury, and systemic hypercoagulability. Subsequent dislodgement of the thrombus tail and its embolization via the systemic venous return into the right heart chambers culminates in impaction within the main pulmonary artery or its segmental branches, thereby manifesting as PE (1, 2). Consequently, PE can be regarded as the terminal progression of venous thrombotic disease and constitutes the predominant clinical phenotype responsible for VTE-related mortality.

The detrimental effects of PE on hemodynamics and gas exchange are characterized by an insidious onset, rapid clinical deterioration, and a markedly elevated case-fatality rate. Acute massive PE precipitates a precipitous increase in right ventricular afterload, culminating in right ventricular failure, a profound reduction in cardiac output, and potentially obstructive shock or cardiac arrest. Even in patients with non-massive PE, recurrent micro-embolic events may engender chronic thromboembolic pulmonary hypertension (CTEPH), thereby severely compromising long-term quality of life (3, 4). Accordingly, early identification and accurate risk stratification of PE remain a core imperative in perioperative critical care management.

Within the diverse etiological spectrum of PE, surgical trauma is unequivocally recognized as one of the most consequential acquired risk factors. Surgical intervention can concurrently activate all three dimensions of Virchow's triad: intraoperative vascular transection and retraction directly compromise endothelial glycocalyx integrity; postoperative immobilization promotes venous stasis in the lower extremities; and the acute-phase response triggered by surgical stress induces a systemic hypercoagulable state (5, 6). Global multicenter observational studies have demonstrated that the overall incidence of postoperative VTE among surgical patients not receiving prophylactic anticoagulation ranges from 15% to 40%, with notably heightened risk observed in patients undergoing major orthopedic procedures and abdominal oncologic surgery (7, 8).

Current clinical instruments for PE assessment predominantly encompass three domains—clinical probability scoring systems, biomarker assays, and imaging modalities—each afflicted by inherent and context-specific limitations.

With respect to clinical probability scoring, although the Wells score and the revised Geneva score are widely endorsed by national and international guidelines, their discriminative validity is substantially attenuated in postoperative cohorts. Given the ubiquitous presence of non-specific findings such as lower-limb edema, immobilization, and hypoxemia in the postoperative setting, numerous scoring components within these instruments become indistinguishable in origin, thereby eroding diagnostic specificity (9).

Regarding biomarker detection, D-dimer serves as a first-line screening tool for PE exclusion in the emergency department owing to its high sensitivity. In the postoperative context, however, surgical wound healing *per se* provokes substantial release of fibrin degradation products, resulting in a precipitous escalation of the false-positive rate and a concomitant decline in specificity to below 30% (10, 11). To circumvent unnecessary radiation exposure and mitigate the risk of contrast-induced nephropathy, there exists an urgent clinical imperative for superior combinatorial biomarker strategies capable of augmenting pretest probability.

Concerning imaging evaluation, computed tomography pulmonary angiography (CTPA) remains the reference standard for confirming PE; nonetheless, its broad application in postoperative patients is constrained by two salient impediments. The first is the substantial economic burden. CTPA entails expenditures related to contrast media, high-pressure injector consumables, and scanner operational time, rendering a single examination a non-negligible financial encumbrance within most healthcare systems. For patients in the postoperative convalescent phase, low-yield CTPA investigations prompted by marginal D-dimer elevations essentially constitute inefficient healthcare resource utilization and an unwarranted escalation of patient financial liability. The second is iatrogenic exposure to ionizing radiation. The effective radiation dose of a single CTPA examination approximates 3–10 mSv, equivalent to several multiples of the annual natural background radiation absorbed by an individual. Cumulative radiation exposure has been empirically linked to a dose–response relationship with the long-term risk of subsequent malignancy. Notably, for younger patients or those with complex postoperative courses necessitating repeated imaging assessments, the long-term oncogenic implications of cumulative radiation exposure are of particular concern. Furthermore, in elderly patients with coexisting postoperative renal insufficiency, the potential hazard of contrast-induced nephropathy further restricts the eligible population for CTPA (12, 13).

In current clinical practice, the prediction of postoperative PE remains largely contingent upon subjective clinician judgment and conventional logistic regression methodologies. The former is circumscribed by individual cognitive biases and divergent clinical experience, whereas the latter, constrained by linearity assumptions, is inherently inadequate for fully delineating the non-linear interactions operative within the postoperative inflammatory-coagulation network. Consequently, the development of an objective quantitative prediction tool capable of integrating dynamic multidimensional postoperative information and accommodating non-linear modeling represents a well-defined and pressing clinical necessity.

In recent years, the advent of machine learning algorithms has provided a novel methodological framework for transcending the limitations inherent to traditional regression models in complex disease prediction. Compared with conventional logistic regression predicated on linearity assumptions, machine learning models—such as random forests, support vector machines, and extreme gradient boosting—possess an intrinsic aptitude for managing high-dimensional non-linear inter-variable relationships (14, 15).

Specifically, the principal advantages of machine learning in the context of postoperative PE prediction manifest at two distinct levels. First, tolerance of variable collinearity: postoperative inflammatory indices (e.g., C-reactive protein, procalcitonin, neutrophil-to-lymphocyte ratio) frequently exhibit high inter-correlation. Under such circumstances, traditional regression models are prone to coefficient estimation bias and variance inflation, whereas ensemble learning algorithms can effectively circumvent this issue through mechanisms of feature importance ranking (16). Second, deep exploration of interaction effects: non-linear interactive phenomena within the postoperative inflammatory-coagulation network (e.g., the modifying effect of elevated NLR on the predictive performance of D-dimer) are challenging to adequately capture using conventional approaches reliant on pre-specified interaction terms, whereas tree-based models and neural network architectures confer a distinct advantage in modeling such complex relationships (17, 18).

Against the backdrop of the aforementioned research context and identified methodological lacunae, the present study endeavors to leverage machine learning algorithms to construct and validate a risk prediction model for PE specifically tailored to postoperative patient populations. The investigation incorporates a multidimensional array of candidate predictors spanning demographic characteristics, surgery-related variables, postoperative inflammatory markers, and coagulation parameters, and systematically compares the performance metrics of multiple supervised learning algorithms in this predictive task. The overarching objective is to substantially enhance the capability for early identification of postoperative PE and to furnish a more precise, individualized, and quantitative evidentiary foundation for clinical decision-making.

## Materials and methods

### Study design and population

This multicenter retrospective study enrolled surgical patients from six tertiary hospitals in China, including Wuxi People's Hospital Affiliated to Nanjing Medical University, Wuxi Second People's Hospital, Yixing People's Hospital, Tengzhou Central People's Hospital, Gaomi People's Hospital, and Tengzhou Hospital of Traditional Chinese Medicine. The study period spanned from January 2020 to January 2025. Based on data provenance, patients from Wuxi People's Hospital Affiliated to Nanjing Medical University, Wuxi Second People's Hospital, and Yixing People's Hospital were assigned to the internal dataset, whereas patients from Tengzhou Central People's Hospital, Gaomi People's Hospital, and Tengzhou Hospital of Traditional Chinese Medicine were reserved as the external validation cohort.

The study enrolled patients aged 18–65 years who underwent surgical procedures and were subsequently diagnosed with postoperative pulmonary embolism confirmed by CTPA or pulmonary angiography. The surgical procedures encompassed multiple subspecialty disciplines, including gastrointestinal surgery (e.g., radical gastrectomy for gastric cancer, radical resection of colorectal cancer, cholecystectomy, appendectomy), urologic surgery (e.g., radical nephrectomy for renal cancer, transurethral resection of the prostate, ureteroscopic lithotripsy), gynecologic surgery (e.g., radical oophorectomy for ovarian cancer, myomectomy, ovarian cystectomy, total hysterectomy), thyroid and breast surgery (e.g., total thyroidectomy, breast lumpectomy), orthopedic surgery (e.g., total hip/knee arthroplasty, internal fixation of hip fractures, spinal fusion), thoracic and cardiovascular surgery (e.g., pulmonary lobectomy, cardiac valve replacement), neurosurgical procedures (e.g., intracranial tumor resection, evacuation of intracranial hematoma), and other superficial procedures (e.g., ophthalmologic, otorhinolaryngologic, and superficial mass excisions). All surgical interventions were performed by senior surgical teams possessing independent operative competency.

Exclusion criteria were as follows: presence of hemorrhagic diathesis (e.g., hemophilia, idiopathic thrombocytopenic purpura), hematologic disorders characterized by abnormalities of the vascular wall or coagulation function; severe cardiac, cerebral, hepatic, or renal insufficiency, psychiatric disorders, or markedly poor compliance; preoperative confirmation of lower extremity deep vein thrombosis or pulmonary embolism; chronic preoperative use of anticoagulant or antiplatelet agents without adequate washout; occurrence of pregnancy, severe infection/sepsis, major hemorrhage, or transfusion of ≥4 units of blood products within 30 days postoperatively; missing data for key predictive variables (including D-dimer, fibrinogen, bedridden status, and surgical type); and patients who underwent a secondary surgical intervention during the same hospital admission.

This retrospective study was approved by the Ethics Committees of all participating institutions, including Wuxi People's Hospital Affiliated to Nanjing Medical University, Wuxi Second People's Hospital, Tengzhou Central People's Hospital, Gaomi People's Hospital, and Tengzhou Hospital of Traditional Chinese Medicine (Approval Nos. KY22086 and IEC-AF/17-1.1). Written informed consent was obtained from all participants, and all personal data were anonymized to safeguard confidentiality.

### Data collection

Data were systematically extracted from the electronic medical record systems of the participating institutions by trained investigators who were blinded to the study outcomes. To ensure temporal consistency and minimize information bias, all variables were collected within prospectively defined perioperative time windows. Preoperative variables were ascertained within 24 h preceding the surgical incision, whereas postoperative laboratory indices were obtained within 48 h following the completion of the surgical procedure.

The following preoperative demographic and clinical characteristics were collected: sex, age, body mass index (BMI), American Society of Anesthesiologists (ASA) physical status classification, serum albumin concentration (ALB), and Nutritional Risk Screening 2002 (NRS2002) score. Lifestyle factors, including history of alcohol consumption and smoking history, were documented based on patient self-report and medical record abstraction. Past medical history variables—comprising prior surgical history, anemia, hypothyroidism, hyperlipidemia, hypertension, diabetes mellitus, chronic obstructive pulmonary disease (COPD), and history of malignancy—were ascertained from preoperative anesthesia evaluations and discharge summaries. Prolonged preoperative bed rest status and occupational exposure (OE) were similarly recorded. Preoperative coagulation parameters, specifically D-dimer and fibrinogen (FIB) concentrations, were obtained from venous blood samples collected within the 24-hour window prior to surgery.

Intraoperative data elements were abstracted from anesthesia records and operative reports. These included: type of anesthesia administered, specific surgical procedure performed, designation of emergency vs. elective surgery, total operative duration, intraoperative blood loss, occurrence of intraoperative hypotension, occurrence of intraoperative tachycardia, nadir intraoperative peripheral oxygen saturation (SpO_2_), and the administration of allogeneic blood transfusion during the intraoperative period.

Postoperative laboratory indices were collected from blood samples drawn within 48 h following the conclusion of surgery. Inflammatory biomarkers included procalcitonin (PCT), C-reactive protein (CRP), serum amyloid A (SAA), and the neutrophil-to-lymphocyte ratio (NLR). Postoperative coagulation parameters, including D-dimer and fibrinogen concentrations, were obtained from the same blood draw where feasible or from the earliest available postoperative sample within the specified 48-hour timeframe.

All laboratory assays were performed in the respective clinical laboratories of the participating centers in accordance with standardized institutional protocols and quality control procedures.

### Definition of postoperative pulmonary embolism and associated factors

Pulmonary embolism refers to a clinical syndrome wherein thrombotic material obstructs the main pulmonary artery or its segmental branches, precipitating hemodynamic compromise, impaired gas exchange, and consequent pulmonary circulatory dysfunction and respiratory insufficiency (4, 19). In the present study, postoperative pulmonary embolism was rigorously defined as an acute pulmonary thromboembolic event objectively confirmed by imaging modalities occurring between the conclusion of the surgical procedure and the stipulated postoperative follow-up interval (e.g., 30 or 90 days postoperatively). To ensure diagnostic accuracy and consistency, this study adopted the imaging findings from CTPA or interventional pulmonary angiography as the core diagnostic reference standard, with supplementary integration of clinical presentation and hemodynamic status to inform a comprehensive adjudication. The specific diagnostic algorithm was executed as follows: an initial clinical evaluation was conducted by the attending physician based on clinical symptomatology (e.g., sudden-onset dyspnea, chest pain, unexplained hypoxemia, or unexplained tachycardia) and pertinent physical signs. For patients with a high clinical pretest probability of PE and hemodynamic stability, CTPA was preferentially performed. Interpretation of CTPA images was independently undertaken by two radiologists with a minimum of five years' experience in thoracic imaging, who explicitly documented the presence of intraluminal filling defects and their precise anatomical location in accordance with internationally established imaging diagnostic criteria. In instances where CTPA findings were equivocal, compromised by technical artifacts, or discordant with a strong clinical suspicion, interventional pulmonary angiography was subsequently pursued to establish a definitive diagnosis (20).

In accordance with prevailing guidelines for the prevention of VTE (21–23), a significant reduction in venous flow velocity can manifest as early as 48 h following surgery or immobilization, thereby escalating the risk of incident VTE. On this basis, the present study operationally defined “prolonged immobilization” as: a postoperative duration of continuous bed rest ≥48 h attributable to pain, fatigue, or monitoring requirements, during which the cumulative duration of out-of-bed activities (including standing and ambulation) was less than 30 min per day.

### Feature selection for model inclusion

To ensure both statistical interpretability and data-driven robustness, a two-stage feature selection strategy was employed to determine the final predictor set for model construction. This framework was designed to integrate conventional regression-based inference with machine learning–based importance ranking, thereby improving the comprehensiveness and stability of variable selection while maintaining clinical interpretability.

Stage 1: Identification of independent risk factors using multivariable logistic regression. In the first stage, all candidate perioperative variables were initially screened using univariate analyses. Variables with statistical significance (*P* < 0.05) were subsequently entered into a multivariable logistic regression model to identify independent predictors of postoperative pulmonary embolism. Variables that remained statistically significant after adjustment were defined as independent risk factors and constituted the regression-based predictor subset with established epidemiological interpretability.

Stage 2: Assessment of feature importance using machine learning algorithms. In the second stage, machine learning models were applied to further evaluate the relative importance and stability of candidate variables under nonlinear and high-dimensional conditions. Five algorithms were constructed, including Extreme Gradient Boosting (XGBoost), Random Forest, Support Vector Machine (SVM), K-Nearest Neighbors (KNN), and Multilayer Perceptron (MLP). For each model, feature importance rankings were generated using model-specific approaches, and the top ten variables with the highest importance scores were extracted from each algorithm.

Final feature set determination: The final set of predictors used for downstream model development was defined as the union of variables identified in Stage 1 (independent risk factors from multivariable logistic regression) and Stage 2 (top-ranked features across machine learning models). This union-based strategy ensured that variables with strong statistical independence as well as those with high predictive contribution in nonlinear models were both retained, thereby improving feature comprehensiveness while preserving interpretability.

This two-stage framework provides a balanced approach between statistical inference and machine learning–based pattern recognition. Logistic regression ensures epidemiological validity and interpretability, whereas machine learning–derived feature ranking enhances sensitivity to complex nonlinear relationships. The integration of both approaches reduces the likelihood of overlooking clinically meaningful predictors and strengthens the robustness of the final modeling dataset.

### Model comparison

The core clinical variables identified through the two-stage progressive feature selection strategy were employed as input features across five distinct machine learning algorithms to construct predictive models for postoperative pulmonary embolism. The objective was to systematically compare the performance characteristics of divergent modeling mechanisms in addressing this specific clinical problem. The algorithms evaluated encompassed mainstream supervised learning paradigms, including Extreme Gradient Boosting (XGBoost), Random Forest, Support Vector Machine (SVM), K-Nearest Neighbors (KNN), and Multilayer Perceptron (MLP). The use of multiple machine learning algorithms (XGBoost, Random Forest, SVM, KNN, and MLP) was not intended to create a “complex ensemble system,” but rather to perform algorithmic benchmarking across different learning paradigms (tree-based, kernel-based, distance-based, and neural network-based methods). This comparative strategy allowed us to identify the most stable and generalizable model under low-event conditions, rather than relying on a single modeling assumption.

Model performance was rigorously evaluated in accordance with established guidelines for predictive model reporting, encompassing three core dimensions: discrimination, calibration, and clinical utility.

Discrimination was quantified by generating Receiver Operating Characteristic (ROC) curves and calculating the Area Under the Curve (AUC) with corresponding 95% confidence intervals (CIs), reflecting the model's capacity to distinguish between patients who did and did not develop the outcome.

Calibration was assessed by constructing calibration curves to visually compare predicted probabilities against actual observed event frequencies. Additionally, the Brier score was calculated to provide a precise measure of the mean squared error between predicted probabilities and binary outcomes, with scores closer to zero indicating superior calibration performance.

Clinical Utility was evaluated using Decision Curve Analysis (DCA). This method calculates the standardized net benefit across a continuum of threshold probabilities, quantifying the incremental value of employing the model for clinical decision-making compared to default strategies of “treat all” or “treat none.” This analysis provides an evidence-based foundation for determining the suitability of model deployment in specific clinical contexts.

### Stratified K-fold cross-validation procedure

To comprehensively evaluate model robustness and generalizability while explicitly addressing the challenges posed by a low-incidence (rare-event) outcome such as postoperative pulmonary embolism, a rigorous stratified k-fold cross-validation procedure was implemented across all five machine learning algorithms. Rather than serving as a purely technical validation step, this resampling strategy was adopted as an essential safeguard against overfitting and optimistic performance estimation, which are particularly common in datasets with highly imbalanced outcome distributions.

Given the relatively low event rate in the present study, a stratified k-fold cross-validation approach was employed to ensure that the proportion of positive and negative cases was preserved within each fold, thereby maintaining consistency with the original dataset distribution. This is critical in rare-event settings, as random partitioning without stratification may lead to unstable training processes and unreliable performance estimates due to insufficient representation of outcome events in certain folds.

Specifically, the original dataset was randomly and stratifiedly divided into k mutually exclusive subsets of approximately equal size (with *k* = 10 in this study). In each iteration, one fold was retained as the validation set, while the remaining *k* − 1 folds were used for model training. This process was repeated k times so that each sample served exactly once as a validation instance. This repeated resampling framework provides a more reliable approximation of model performance than a single train–test split, particularly in the context of limited event numbers, where performance estimates are highly sensitive to data partitioning.

The overall predictive performance of each model was obtained by averaging the evaluation metrics across all k iterations. This averaging process reduces variance introduced by random sampling variability and yields a more stable and approximately unbiased estimate of model generalization performance. Importantly, in the context of rare-event modeling, such cross-validation is not merely a technical optimization step but a critical methodological safeguard to ensure that observed performance is not driven by idiosyncratic characteristics of a single data split.

This uniform stratified cross-validation framework was consistently applied to XGBoost, Random Forest, SVM, KNN, and MLP models, ensuring that comparisons among different algorithmic architectures were performed on a standardized and fair basis. The primary objective of implementing k-fold cross-validation in this study was twofold (1): to mitigate overfitting to noise, outliers, and dataset-specific patterns that are more pronounced in small-event scenarios; and (2) to evaluate model stability under repeated perturbations of the training distribution, thereby providing a more reliable assessment of robustness in real-world clinical settings.

For each validation fold, the AUC was calculated as the primary discrimination metric. The final cross-validation performance was reported as the mean ± standard deviation of AUC values across all folds, reflecting both central tendency and variability, which together provide a comprehensive assessment of model accuracy and stability under rare-event conditions.

### Supplementary stability assessment and classification performance validation

Beyond conventional discrimination and calibration analyses, this study implemented supplementary assessments of model stability and classification performance.

First, learning curves were constructed to visualize the dynamic evolution of model performance as a function of increasing training sample size. The training set was subsampled at incremental proportions (e.g., from 10% to 100% of the total training data). At each subsample scale, cross-validation was repeated to calculate performance metrics on both the training and validation subsets. Observation of the convergence trajectory and the relative gap between the training and validation performance curves allowed for a systematic evaluation of potential overfitting (where training performance markedly exceeds validation performance with a persistently wide margin) or underfitting (where both curves plateau at suboptimal performance levels), thereby informing judgments regarding the adequacy of current model complexity relative to sample size.

Second, the Kolmogorov–Smirnov (KS) statistic was introduced as a supplementary metric for evaluating risk stratification capacity. The KS statistic is defined as the maximum vertical distance between the cumulative distribution functions of predicted probabilities assigned to positive and negative samples. Its value ranges from 0 to 1, with higher values denoting superior discriminatory power. KS curves were further plotted to pinpoint the threshold corresponding to maximum separation between the cumulative distribution curves, offering a reference for determining optimal classification cut-off values.

Third, to assess performance in real-world binary classification scenarios, confusion matrices were constructed for both the training and validation sets based on the optimal classification threshold determined by maximizing the Youden Index. These matrices precisely detail the frequencies of True Positives (TP), True Negatives (TN), False Positives (FP), and False Negatives (FN). Comparison of the discrepancies in derived classification metrics between the training and validation sets enabled an effective assessment of classification consistency across different data partitions. Substantial disparity in these metrics would indicate a propensity for overfitting, warranting cautious interpretation of generalizability.

### External validation for generalizability assessment

To further evaluate the generalizability and cross-scenario clinical applicability of the final selected predictive model, a rigorous external validation was conducted using an independently collected multi-center external dataset. This external cohort was entirely independent of the model development cohort with respect to temporal span, geographic region, and tier of healthcare institution, thereby enabling a robust assessment of model robustness and transportability across diverse clinical practice patterns, patient demographics, and diagnostic-therapeutic contexts.

During this validation phase, the finalized prediction model was applied to the external dataset in a completely frozen state. That is, the model architecture, feature weights, and all hyperparameter configurations remained entirely unaltered from the internal training phase, with no form of retraining, fine-tuning, or calibration update performed on the external data. This stringent procedure was intended to faithfully reflect the model's actual generalization performance when removed from its original developmental environment.

The predictive performance on the external validation set was systematically scrutinized adhering to the established three-dimensional evaluation framework of discrimination, calibration, and clinical utility. For discrimination assessment, the ROC curve was plotted for the external validation cohort, and the AUC with its 95% CI was computed. For calibration assessment, a calibration curve specific to the external validation set was generated to visually examine the congruence between predicted probabilities and observed outcome incidence. For clinical utility assessment, Decision Curve Analysis was performed independently on the external validation set.

### Model interpretability

To overcome the inherent “black-box” limitation of machine learning models and enhance clinical interpretability, this study adopted a dual interpretability framework that integrates SHapley Additive exPlanations (SHAP) for the machine learning model and a nomogram for the logistic regression model. This complementary strategy was designed to provide both global and individualized interpretations of predictive factors across different modeling paradigms. For the machine learning-based model, SHAP analysis was performed to quantify the contribution of each predictor to the final model output. Grounded in cooperative game theory, SHAP values represent the marginal contribution of each feature to the predicted risk of postoperative pulmonary embolism, enabling consistent attribution of feature importance across the entire dataset.

At the global level, SHAP summary plots were constructed to rank variables by their mean absolute SHAP values, thereby identifying the most influential predictors in the model. Additionally, the directionality of each predictor was visualized using color-coded feature distributions, allowing for simultaneous assessment of variable magnitude and its association with predicted risk. At the individual level, SHAP decision plots were generated for single patients to illustrate how each clinical variable incrementally contributes to the final predicted probability. By showing how each feature increases or decreases the baseline risk, these plots enable case-specific decomposition of risk predictions, thereby enhancing the transparency of individualized risk estimation.

Meanwhile, to provide a clinically intuitive and easily applicable risk assessment tool, a nomogram was developed based on the final multivariable logistic regression model. All independent predictors identified in this study were incorporated into the nomogram. Each predictor was assigned a weighted score proportional to its regression coefficient, and individual patient values were mapped onto a "Points" scale (ranging from 0 to 100). The scores for all predictors were then summed to obtain a total score, which was subsequently converted into an individualized probability of postoperative pulmonary embolism using the risk scale at the bottom of the nomogram. This allows for bedside risk assessment without the need for computational tools.

## Results

### Baseline clinical characteristics of the enrolled cohort

**Figure 1:**
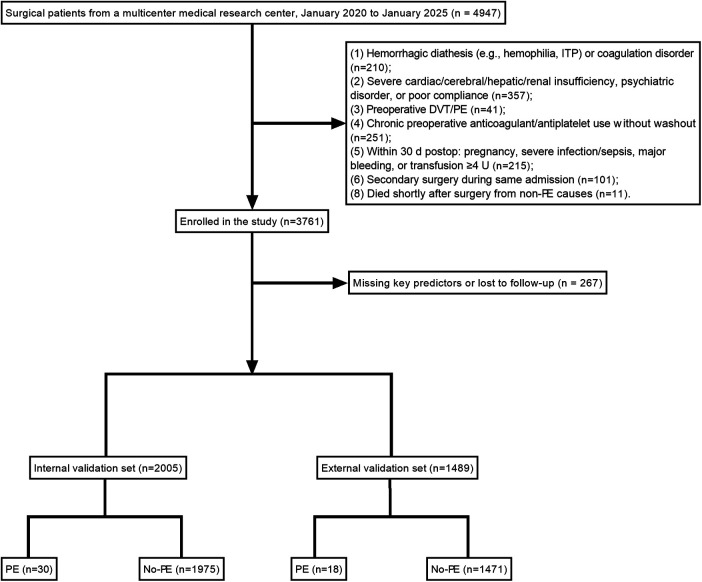
Flow diagram depicting the study selection cascade in accordance with the predefined inclusion and exclusion criteria.

A total of 3,494 patients were included in this study, comprising 2,005 patients in the internal dataset and 1,489 patients in the external validation cohort. Postoperative PE occurred in 30 patients (1.50%) in the internal dataset and 18 patients (1.21%) in the external validation cohort, respectively (Figure 1).

Compared with the non-PE group, patients who developed PE were more likely to be older (≥65 years: 62.5% vs. 43.4%, *P* = 0.013) and had a higher body mass index (BMI ≥25 kg/m^2^: 66.7% vs. 36.7%, *P* < 0.001). In addition, hypoalbuminemia (ALB < 30 g/L) was more prevalent in the PE group (45.8% vs. 29.8%, *P* = 0.024). Regarding comorbidities, hypertension (45.8% vs. 17.9%, *P* < 0.001), diabetes (35.4% vs. 14.0%, *P* < 0.001), and a history of malignancy (66.7% vs. 21.4%, *P* < 0.001) were significantly more common in patients with PE. Prolonged bed rest was also strongly associated with PE occurrence (62.5% vs. 24.5%, *P* < 0.001). In terms of perioperative factors, emergency surgery (45.8% vs. 28.6%, *P* = 0.014), longer operative duration (≥270 min: 66.7% vs. 28.0%, *P* < 0.001), and intraoperative tachycardia (60.4% vs. 24.6%, *P* < 0.001) were significantly more frequent in the PE group. General anesthesia was less commonly used in patients with PE (83.3% vs. 92.6%, *P* = 0.025). For laboratory parameters, elevated preoperative D-dimer (≥0.5 mg/L: 45.8% vs. 23.7%, *P* = 0.001) was more common in the PE group. Postoperatively, inflammatory markers including procalcitonin (PCT ≥0.05 ng/mL: 60.4% vs. 23.2%, *P* < 0.001), C-reactive protein (CRP ≥10 mg/L: 64.6% vs. 21.9%, *P* < 0.001), and neutrophil-to-lymphocyte ratio (NLR ≥3: 66.7% vs. 25.7%, *P* < 0.001) were significantly elevated in patients with PE. Notably, postoperative D-dimer levels were markedly higher in the PE group (≥0.5 mg/L: 79.2% vs. 28.6%, *P* < 0.001). No significant differences were observed between the two groups in sex, ASA classification, nutritional risk score, smoking or drinking history, most comorbidities, surgical category, intraoperative hypotension, oxygen saturation, blood transfusion, or postoperative fibrinogen levels (all *P* > 0.05) (Table 1). The original dataset utilized in this study is provided in Supplementary Table S1.

**Table 1:** Baseline clinical characteristics in the dataset.

variable classification		All (*n* = 3,494)	Non-PE (*n* = 3,446)	PE (*n* = 48)	*P*-value
Sex	Female	1,820 (52.089%)	1,798 (52.176%)	22 (45.833%)	0.467
	Male	1,674 (47.911%)	1,648 (47.824%)	26 (54.167%)	
Age	<65	1,967 (56.297%)	1,949 (56.558%)	18 (37.500%)	0.013
	≥65	1,527 (43.703%)	1,497 (43.442%)	30 (62.500%)	
BMI	<25 kg/m^2^	2,197 (62.879%)	2,181 (63.291%)	16 (33.333%)	<0.001
	≥25 kg/m^2^	1,297 (37.121%)	1,265 (36.709%)	32 (66.667%)	
ASA	<3	2,497 (71.465%)	2,465 (71.532%)	32 (66.667%)	0.562
	≥3	997 (28.535%)	981 (28.468%)	16 (33.333%)	
ALB	≥30g/L	2,446 (70.006%)	2,420 (70.226%)	26 (54.167%)	0.024
	<30g/L	1,048 (29.994%)	1,026 (29.774%)	22 (45.833%)	
NRS2002 score	<3	2,437 (69.748%)	2,402 (69.704%)	35 (72.917%)	0.747
	≥3	1,057 (30.252%)	1,044 (30.296%)	13 (27.083%)	
Drinking history	No	2,296 (65.713%)	2,267 (65.786%)	29 (60.417%)	0.532
	Yes	1,198 (34.287%)	1,179 (34.214%)	19 (39.583%)	
Smoking history	No	2,509 (71.809%)	2,471 (71.706%)	38 (79.167%)	0.327
	Yes	985 (28.191%)	975 (28.294%)	10 (20.833%)	
Surgical history	No	2,725 (77.991%)	2,687 (77.974%)	38 (79.167%)	0.982
	Yes	769 (22.009%)	759 (22.026%)	10 (20.833%)	
Anemia	No	3,053 (87.378%)	3,014 (87.464%)	39 (81.250%)	0.285
	Yes	441 (12.622%)	432 (12.536%)	9 (18.750%)	
Hypothyroidism	No	2,760 (78.993%)	2,724 (79.048%)	36 (75.000%)	0.613
	Yes	734 (21.007%)	722 (20.952%)	12 (25.000%)	
Hyperlipidemia	No	2,840 (81.282%)	2,806 (81.428%)	34 (70.833%)	0.092
	Yes	654 (18.718%)	640 (18.572%)	14 (29.167%)	
Hypertension	No	2,855 (81.712%)	2,829 (82.095%)	26 (54.167%)	<0.001
	Yes	639 (18.288%)	617 (17.905%)	22 (45.833%)	
Diabetes	No	2,996 (85.747%)	2,965 (86.042%)	31 (64.583%)	<0.001
	Yes	498 (14.253%)	481 (13.958%)	17 (35.417%)	
COPD	No	3,125 (89.439%)	3,083 (89.466%)	42 (87.500%)	0.839
	Yes	369 (10.561%)	363 (10.534%)	6 (12.500%)	
Malignancy history	No	2,726 (78.019%)	2,710 (78.642%)	16 (33.333%)	<0.001
	Yes	768 (21.981%)	736 (21.358%)	32 (66.667%)	
Prolonged bed rest	No	2,621 (75.014%)	2,603 (75.537%)	18 (37.500%)	<0.001
	Yes	873 (24.986%)	843 (24.463%)	30 (62.500%)	
OE	No	2,368 (67.773%)	2,338 (67.847%)	30 (62.500%)	0.528
	Yes	1,126 (32.227%)	1,108 (32.153%)	18 (37.500%)	
Preoperative D-dimer	<0.5 mg/L	2,656 (76.016%)	2,630 (76.320%)	26 (54.167%)	0.001
	≥0.5 mg/L	838 (23.984%)	816 (23.680%)	22 (45.833%)	
Preoperative FIB	<4.0 g/L	2,678 (76.646%)	2,645 (76.756%)	33 (68.750%)	0.258
	≥4.0 g/L	816 (23.354%)	801 (23.244%)	15 (31.250%)	
Anesthesia type	General anesthesia	3,231 (92.473%)	3,191 (92.600%)	40 (83.333%)	0.025
	Regional anesthesia	263 (7.527%)	255 (7.400%)	8 (16.667%)	
Surgical procedure	Digestive System Surgery	964 (27.590)	943 (27.365)	21 (43.750)	0.396
	Urinary System Surgery	352 (10.074)	348 (10.099)	4 (8.333)	
	Gynecological Surgery	391 (11.191)	386 (11.201)	5 (10.417)	
	Thyroid/Breast Surgery	394 (11.276)	391 (11.346)	3 (6.250)	
	Orthopedic Surgery	663 (18.975)	657 (19.066)	6 (12.500)	
	Thoracic/Cardiovascular Surgery	390 (11.162)	386 (11.201)	4 (8.333)	
	Neurosurgery	209 (5.982)	206 (5.978)	3 (6.250)	
	Other Superficial Surgeries	131 (3.749)	129 (3.743)	2 (4.167)	
Emergency surgery	No	2,488 (71.208%)	2,462 (71.445%)	26 (54.167%)	0.014
	Yes	1,006 (28.792%)	984 (28.555%)	22 (45.833%)	
Surgery time	<270 min	2,497 (71.465%)	2,481 (71.997%)	16 (33.333%)	<0.001
	≥270 min	997 (28.535%)	965 (28.003%)	32 (66.667%)	
Intraoperative bleeding	<100 mL	2,521 (72.152%)	2,492 (72.316%)	29 (60.417%)	0.096
	≥100 mL	973 (27.848%)	954 (27.684%)	19 (39.583%)	
Intraoperative hypotension	No	2,593 (74.213%)	2,560 (74.289%)	33 (68.750%)	0.481
	Yes	901 (25.787%)	886 (25.711%)	15 (31.250%)	
Intraoperative tachycardia	No	2,619 (74.957%)	2,600 (75.450%)	19 (39.583%)	<0.001
	Yes	875 (25.043%)	846 (24.550%)	29 (60.417%)	
Intraoperative SpO_2_	≥90%	2,960 (84.717%)	2,920 (84.736%)	40 (83.333%)	0.947
	<90%	534 (15.283%)	526 (15.264%)	8 (16.667%)	
Blood transfusion	No	3,213 (91.958%)	3,169 (91.962%)	44 (91.667%)	0.792
	Yes	281 (8.042%)	277 (8.038%)	4 (8.333%)	
PCT level	<0.05 ng/mL	2,667 (76.331%)	2,648 (76.843%)	19 (39.583%)	<0.001
	≥0.05 ng/mL	827 (23.669%)	798 (23.157%)	29 (60.417%)	
CRP level	<10 mg/L	2,710 (77.562%)	2,693 (78.149%)	17 (35.417%)	<0.001
	≥10 mg/L	784 (22.438%)	753 (21.851%)	31 (64.583%)	
SAA level	<10 mg/L	2,643 (75.644%)	2,611 (75.769%)	32 (66.667%)	0.197
	≥10 mg/L	851 (24.356%)	835 (24.231%)	16 (33.333%)	
NLR	<3	2,578 (73.784%)	2,562 (74.347%)	16 (33.333%)	<0.001
	≥3	916 (26.216%)	884 (25.653%)	32 (66.667%)	
Postoperative D-dimer	<0.5 mg/L	2,471 (70.721%)	2,461 (71.416%)	10 (20.833%)	<0.001
	≥0.5 mg/L	1,023 (29.279%)	985 (28.584%)	38 (79.167%)	
Postoperative FIB	<4.0 g/L	2,492 (71.322%)	2,455 (71.242%)	37 (77.083%)	0.467
	≥4.0 g/L	1,002 (28.678%)	991 (28.758%)	11 (22.917%)	

OR, odds ratio; CI, confidence interval; BMI, body mass index; ASA, The American Society of Anesthesiologists; ALB, albumin; PCT, procalcitonin; CRP, C-reactive protein; SAA, serum amyloid A; NRS2,002, nutrition risk screening 2002; COPD, chronic obstructive pulmonary disease; SPO_2_, percutaneous arterial oxygen saturation; OE, occupational exposure; FIB, fibrinogen.

### Multivariable logistic regression and machine learning algorithms as traditional statistical benchmarks for identifying independent risk factors

Variable selection was performed using a combination of traditional statistical analysis and machine learning approaches.

In the univariate analysis, age, BMI, hypertension, diabetes, malignancy history, prolonged bed rest, anesthesia type, surgery time, intraoperative tachycardia, PCT level, CRP level, NLR, and postoperative D-dimer were significantly associated with postoperative pulmonary embolism (all *P* < 0.05).

Subsequent multivariate logistic regression analysis identified age, BMI, hypertension, malignancy history, prolonged bed rest, surgery time, intraoperative tachycardia, CRP level, NLR, and postoperative D-dimer as independent predictors of postoperative PE (Table 2).

**Table 2:** Univariate and multivariate logistic regression analysis for postoperative PE.

Variables		Univariate analysis		Multivariate analysis	
		OR, 95%CI	*P*-value	OR, 95%CI	*P*-value
Sex	Female	Reference			
	Male	1.921[0.910,4.058]	0.087		
Age	<65	Reference		Reference	
	≥65	2.725[1.269,5.852]	0.01	2.480[1.064,6.159]	0.041
BMI	<25 kg/m^2^	Reference		Reference	
	≥25 kg/m^2^	3.192[1.455,7.005]	0.004	2.677[1.127,6.893]	0.031
ASA	<3	Reference			
	≥3	0.51[0.194,1.340]	0.172		
ALB	≥30g/L	Reference			
	<30g/L	1.94[0.941,3.999]	0.073		
NRS2002 score	<3	Reference			
	≥3	0.691[0.295,1.619]	0.395		
Drinking history	No	Reference			
	Yes	0.877[0.408,1.885]	0.737		
Smoking history	No	Reference			
	Yes	0.708[0.302,1.658]	0.426		
Surgical history	No	Reference			
	Yes	1.276[0.564,2.886]	0.558		
Anemia	No	Reference			
	Yes	1.874[0.796,4.408]	0.15		
Hypothyroidism	No	Reference			
	Yes	1.396[0.635,3.068]	0.407		
Hyperlipidemia	No	Reference			
	Yes	1.014[0.432,2.378]	0.975		
Hypertension	No	Reference		Reference	
	Yes	2.622[1.264,5.439]	0.01	2.658[1.021,6.769]	0.041
Diabetes	No	Reference		Reference	
	Yes	2.47[1.120,5.446]	0.025	1.066[0.363,2.905]	0.904
COPD	No	Reference			
	Yes	0.97[0.292,3.225]	0.96		
Malignancy history	No	Reference		Reference	
	Yes	6.876[3.195,14.798]	<0.001	2.811[1.199,6.853]	0.019
Prolonged bed rest	No	Reference		Reference	
	Yes	4.771[2.255,10.092]	<0.001	2.639[1.149,6.270]	0.024
OE	No	Reference			
	Yes	0.789[0.367,1.695]	0.544		
Preoperative D-dimer	<0.5 mg/L	Reference			
	≥0.5 mg/L	2.048[0.980,4.282]	0.057		
Preoperative FIB	<4.0 g/L	Reference			
	≥4.0 g/L	1.967[0.941,4.111]	0.072		
Anesthesia type	General anesthesia	Reference		Reference	
	Regional anesthesia	2.662[1.004,7.063]	0.049	2.712[0.769,8.214]	0.093
Surgical procedure	Digestive System Surgery	Reference			
	Urinary System Surgery	0.362[0.082,1.597]	0.18		
	Gynecological Surgery	0.509[0.146,1.776]	0.29		
	Thyroid/Breast Surgery	0.187[0.025,1.427]	0.106		
	Orthopedic Surgery	0.412[0.136,1.251]	0.117		
	Thoracic/Cardiovascular Surgery	0.373[0.084,1.644]	0.192		
	Neurosurgery	0.721[0.162,3.198]	0.667		
	Other Superficial Surgeries	0.477[0.062,3.664]	0.477		
Emergency surgery	No	Reference			
	Yes	1.835[0.885,3.802]	0.103		
Surgery time	<270 min	Reference		Reference	
	≥270 min	3.637[1.720,7.687]	0.001	3.133[1.380,7.446]	0.007
Intraoperative bleeding	<100 mL	Reference			
	≥100 mL	1.208[0.571,2.553]	0.621		
Intraoperative hypotension	No	Reference			
	Yes	0.692[0.281,1.703]	0.423		
Intraoperative tachycardia	No	Reference		Reference	
	Yes	6.097[2.880,12.909]	<0.001	3.767[1.658,8.919]	0.002
Intraoperative SpO_2_	≥90%	Reference			
	<90%	1.924[0.728,5.085]	0.187		
Blood transfusion	No	Reference			
	Yes	0.81[0.191,3.432]	0.775		
PCT level	<0.05 ng/mL	Reference		Reference	
	≥0.05 ng/mL	6.045[2.810,13.002]	<0.001	2.312[0.992,5.614]	0.056
CRP level	<10 mg/L	Reference		Reference	
	≥10 mg/L	7.018[3.261,15.105]	<0.001	2.637[1.115,6.518]	0.03
SAA level	<10 mg/L	Reference			
	≥10 mg/L	1.328[0.604,2.918]	0.481		
NLR	<3	Reference		Reference	
	≥3	5.235[2.474,11.077]	<0.001	2.839[1.228,6.839]	0.016
Postoperative D-dimer	<0.5 mg/L	Reference		Reference	
	≥0.5 mg/L	6.534[2.892,14.761]	<0.001	3.034[1.277,7.838]	0.015
Postoperative FIB	<4.0 g/L	Reference			
	≥4.0 g/L	0.565[0.230,1.389]	0.213		

OR, odds ratio; CI, confidence interval; BMI, body mass index; ASA, The American Society of Anesthesiologists; ALB, albumin; PCT, procalcitonin; CRP, C-reactive protein; SAA, serum amyloid A; NRS2002, nutrition risk screening 2002; COPD, chronic obstructive pulmonary disease; SPO_2_, percutaneous arterial oxygen saturation; OE, occupational exposure; FIB, fibrinogen.

**Figure 2:**
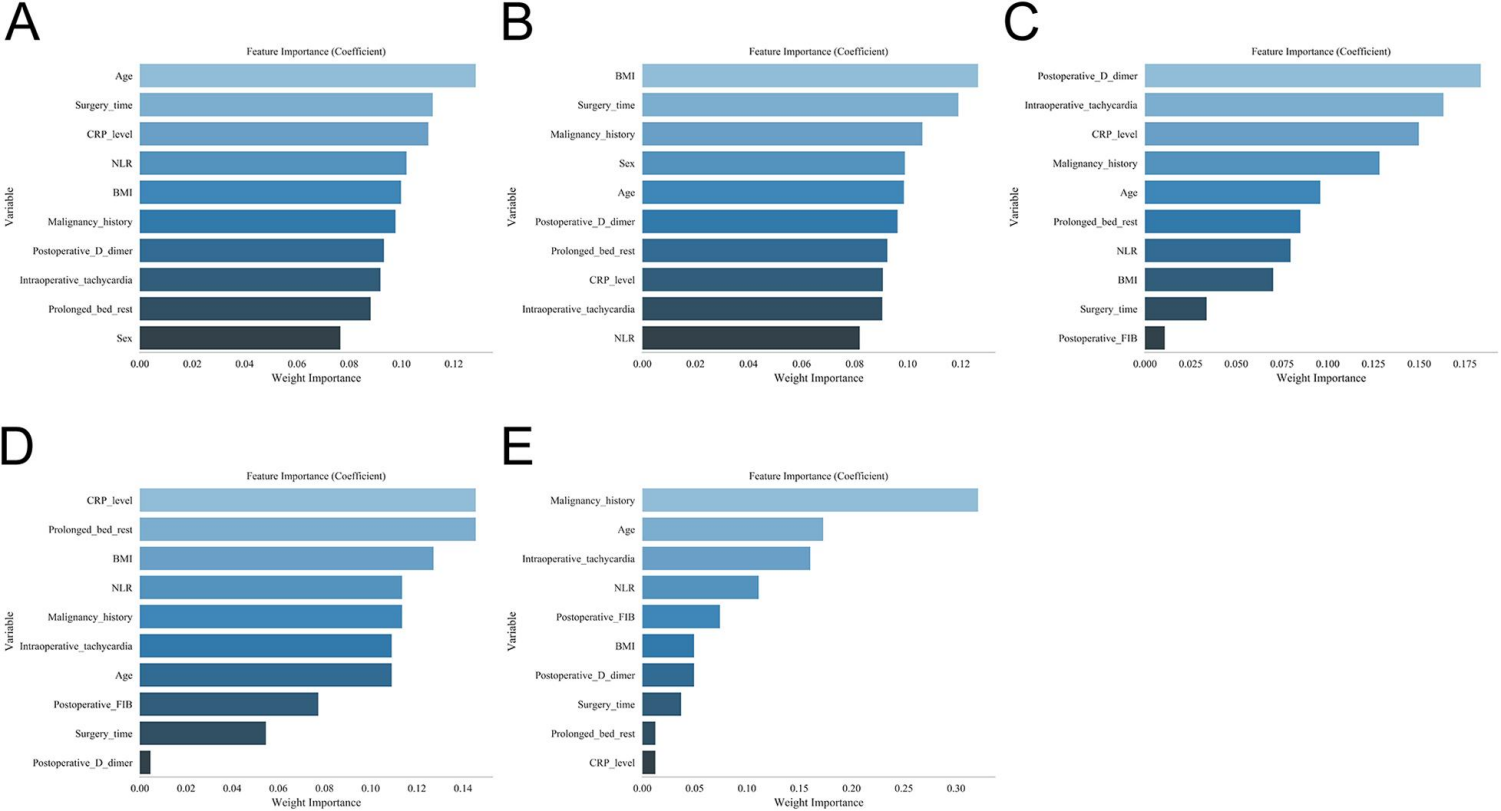
Feature importance ranking for the prediction of postoperative pulmonary embolism derived from five distinct machine learning algorithms. **(A)** XGBoost, **(B)** Random Forest, **(C)** Support Vector Machine, **(D)** K-Nearest Neighbors, and **(E)** Multilayer Perceptron.

To further enhance feature robustness, five machine learning models, including XGBoost, RF, SVM, MLP, and KNN, were applied for feature importance ranking. The results consistently demonstrated that age, BMI, malignancy history, prolonged bed rest, surgery time, intraoperative tachycardia, CRP level, NLR, and postoperative D-dimer were ranked among the top ten features across all five models (Figures 2A–E).

Based on the combined criteria of statistical significance and model stability, the final feature set included age, BMI, malignancy history, prolonged bed rest, surgery time, intraoperative tachycardia, CRP level, and NLR.

### XGBoost as the primary machine learning model demonstrating the best overall performance

All selected variables were incorporated into five machine learning models, including KNN, XGBoost, RF, SVM, and MLP, and their predictive performances were compared. Receiver operating characteristic (ROC) curve analysis demonstrated that the XGBoost model achieved the best discriminative performance among all models. Among the five evaluated machine learning models, performance comparison across multiple metrics, including AUC, sensitivity, specificity, F1 score, and Cohen's Kappa, demonstrated that XGBoost achieved the most balanced and robust predictive performance in both the training and validation cohorts.

**Figure 3:**
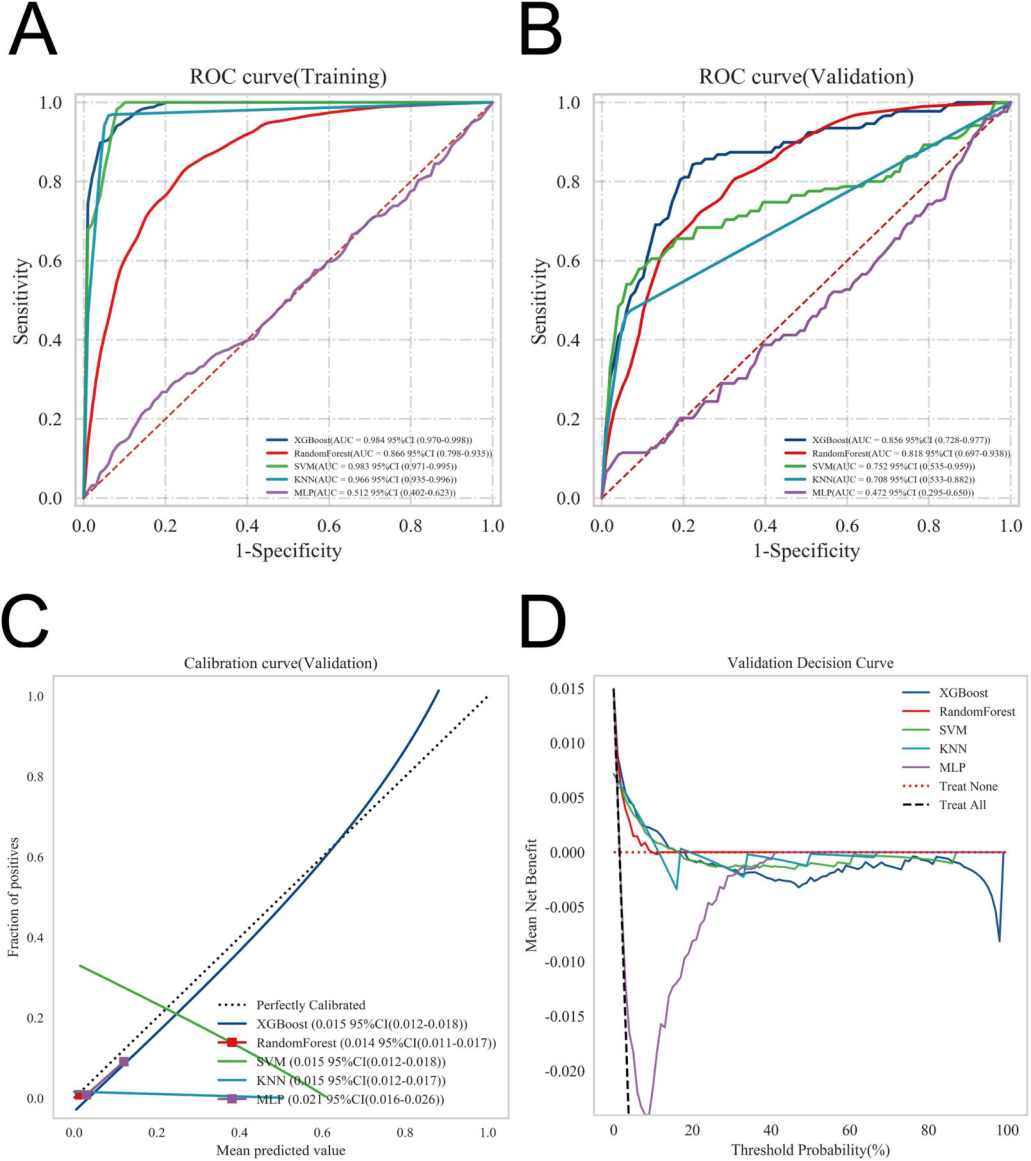
Comparative assessment of predictive performance, model stability, and clinical utility across five machine learning algorithms. **(A)** Receiver operating characteristic (ROC) curves for the training cohort, illustrating the discriminative capacity of each model. **(B)** ROC curves for the validation cohort, reflecting model generalizability to unseen data. **(C)** Calibration curves depicting the concordance between model-predicted probabilities and empirically observed outcome frequencies. **(D)** Decision curve analysis (DCA) quantifying the standardized net benefit across a continuum of risk threshold probabilities to appraise the clinical utility of each candidate model.

In the validation set, XGBoost yielded an AUC of 0.856 (95% CI: 0.728–0.977), with a sensitivity of 0.583 (95% CI: 0.439–0.726), specificity of 0.915 (95% CI: 0.893–0.936), F1 score of 0.841 (95% CI: 0.722–0.961), and Kappa of 0.784 (95% CI: 0.623–0.946). Compared with other models, XGBoost demonstrated a more favorable balance between sensitivity and specificity, particularly maintaining higher sensitivity than KNN and SVM in the validation cohort, which showed substantially reduced sensitivity (0.467 and 0.557, respectively) despite relatively high specificity. Random Forest showed stable but comparatively lower overall agreement (Kappa: 0.452), with reduced F1 score and weaker discriminative balance between classes. SVM and KNN exhibited acceptable specificity but significantly compromised sensitivity in the validation cohort, indicating a tendency toward majority-class classification and reduced detection capability for postoperative pulmonary embolism cases. Although both models achieved high AUC values in the training set, their reduced sensitivity in external data limited their clinical applicability in imbalanced outcome settings. The MLP model demonstrated consistently poor performance across all evaluation metrics in both training and validation cohorts, indicating inadequate learning of discriminative patterns in the present dataset. Taken together, although several models exhibited comparable AUC values, XGBoost was selected as the final model due to its superior balance across discrimination, calibration-related classification stability (Kappa), and clinically relevant sensitivity–specificity trade-off in the validation cohort. This comprehensive evaluation supports its robustness and suitability for further external validation and clinical interpretation in the context of rare-event postoperative pulmonary embolism prediction (Table 3, Figure 3A).

**Table 3:** Comparison of selected features across five machine learning models.

Machine learning	Classification	AUC (95%CI)	Accuracy (95%CI)	Sensitivity (95%CI)	Specificity (95%CI)	F1 score (95%CI)	Kappa (95%CI)
KNN	training set	0.966 (0.935–0.996)	0.949 (0.945–0.953)	0.969 (0.950–0.988)	0.949 (0.945–0.953)	0.883 (0.876–0.890)	0.839 (0.831–0.846)
	validation set	0.708 (0.533–0.882)	0.937 (0.932–0.941)	0.467 (0.379–0.555)	0.944 (0.939–0.949)	0.814 (0.787–0.841)	0.743 (0.709–0.777)
XGBoost	training set	0.984 (0.970–0.998)	0.917 (0.893–0.940)	0.965 (0.944–0.985)	0.916 (0.892–0.940)	0.96 (0.955–0.965)	0.946 (0.940–0.952)
	validation set	0.856 (0.728–0.977)	0.91 (0.890–0.930)	0.583 (0.439–0.726)	0.915 (0.893–0.936)	0.841 (0.722–0.961)	0.784 (0.623–0.946)
RF	training set	0.866 (0.798–0.935)	0.776 (0.733–0.819)	0.844 (0.793–0.894)	0.775 (0.731–0.819)	0.646 (0.635–0.657)	0.486 (0.472–0.500)
	validation set	0.818 (0.697–0.938)	0.779 (0.741–0.817)	0.725 (0.595–0.856)	0.78 (0.740–0.821)	0.626 (0.582–0.669)	0.452 (0.400–0.504)
SVM	training set	0.983 (0.971–0.995)	0.926 (0.918–0.934)	0.982 (0.977–0.991)	0.925 (0.917–0.933)	0.968 (0.948–0.987)	0.957 (0.931–0.983)
	validation set	0.752 (0.535–0.959)	0.925 (0.915–0.934)	0.557 (0.468–0.646)	0.931 (0.920–0.942)	0.796 (0.689–0.903)	0.717 (0.568–0.865)
MLP	training set	0.512 (0.402–0.623)	0.429 (0.207–0.651)	0.793 (0.600–0.986)	0.424 (0.196–0.651)	0.749 (0.640–0.859)	0.654 (0.481–0.827)
	validation set	0.472 (0.295–0.650)	0.409 (0.192–0.625)	0.623 (0.397–0.849)	0.405 (0.182–0.628)	0.755(0.719–0.791)	0.662(0.587–0.736)

CI, confidence interval; KNN, k-nearest neighbor; XGBoost, extreme gradient boosting; RF, random forest; SV*M*, support vector machine; MLP, multilayer perceptron; AUC, area under the curve.

Calibration curve analysis indicated that XGBoost and Random Forest demonstrated good agreement between predicted and observed outcomes, whereas SVM and KNN showed relatively poor calibration. The Brier scores further supported these findings, with lower values observed for Random Forest (0.014, 95% CI: 0.011–0.017) and XGBoost (0.015, 95% CI: 0.012–0.018), compared to higher values for MLP (0.021, 95% CI: 0.016–0.026) (Figure 3B).

Decision curve analysis (DCA) revealed that XGBoost and Random Forest provided superior net clinical benefit across a wide range of threshold probabilities, whereas the MLP model demonstrated limited clinical utility (Figure 3C).

**Figure 4:**
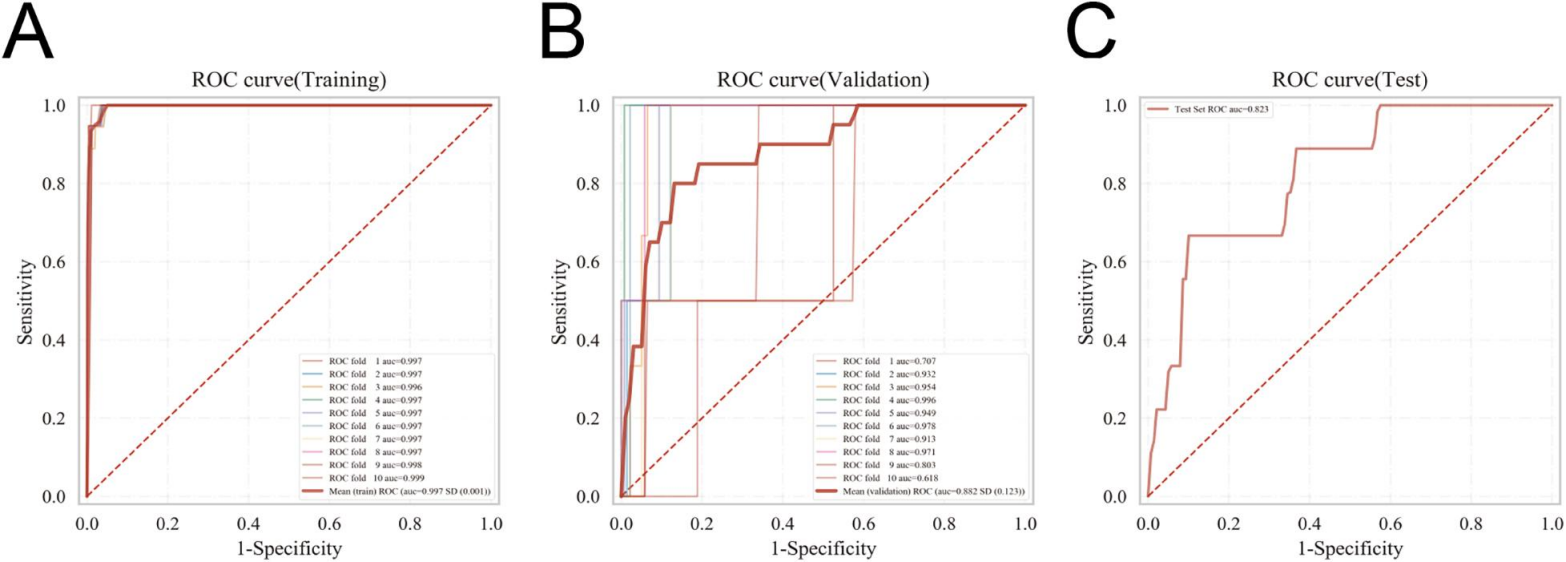
K-fold cross-validation performance of the XGBoost model. The robustness and generalizability of the XGBoost algorithm were systematically evaluated via k-fold cross-validation. **(A)** ROC curves for the training folds across all iterations, demonstrating internal consistency. **(B)** ROC curves for the validation folds, illustrating model stability during the hyperparameter optimization phase. **(C)** ROC curves for the test folds, reflecting the model's predictive fidelity on previously unseen data partitions.

To further evaluate model generalizability, a k-fold cross-validation strategy was applied. The entire dataset was randomly divided into a training set and a testing set, with 602 patients (30.02%) allocated to the testing set and the remaining samples used for 10-fold cross-validation. Among the five models, the XGBoost model demonstrated the best overall performance and stability, achieving a mean AUC of 0.8821 ± 0.1226 in cross-validation. In the independent testing set, XGBoost maintained robust predictive performance with an AUC of 0.8232 and an accuracy of 0.9419 (Figures 4A–C). The RF model showed moderate performance, with a cross-validation AUC of 0.7834 ± 0.1775 and a testing AUC of 0.7266 (accuracy: 0.9102). The SVM and KNN models demonstrated relatively high variability across folds (AUC: 0.8381 ± 0.1927 and 0.8328 ± 0.1586, respectively), suggesting limited stability despite acceptable performance in the testing set (AUC: 0.7464 and 0.8135, respectively). The MLP model exhibited relatively stable but lower performance, with a cross-validation AUC of 0.7502 ± 0.0518 and a testing AUC of 0.7575 (accuracy: 0.8150).

**Figure 5:**
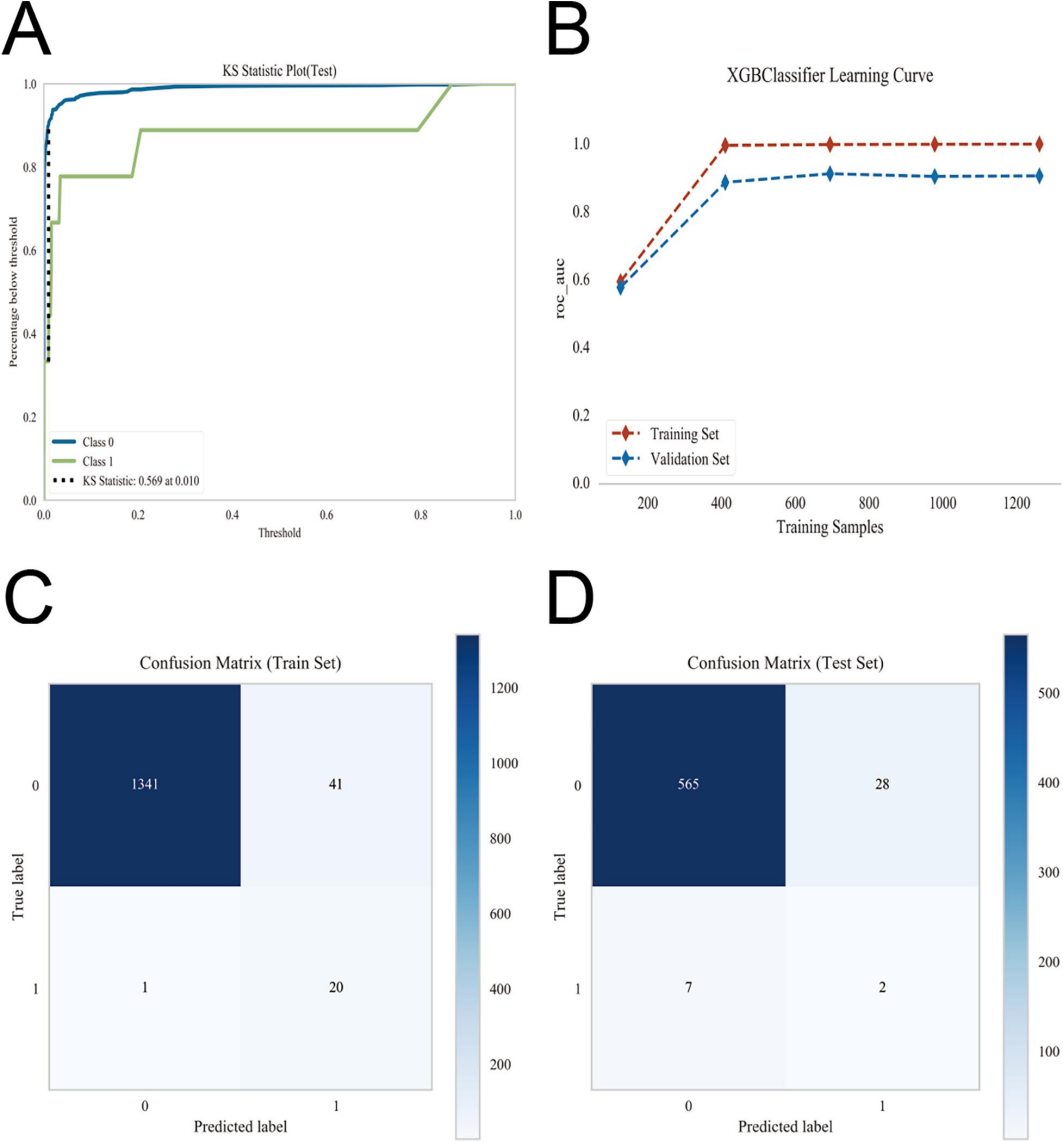
Multidimensional performance evaluation of the XGBoost model encompassing discrimination, learning dynamics, and classification fidelity. **(A)** Kolmogorov-Smirnov (KS) curve depicting the maximal divergence between the cumulative distribution functions of positive and negative outcome classes, serving as an index of discriminative power. **(B)** Learning curve illustrating the trajectory of training and validation performance as a function of training iterations, thereby enabling assessment of model fitting and detection of potential overfitting or underfitting. **(C)** Confusion matrix derived from the training set, detailing the frequencies of true positives, true negatives, false positives, and false negatives. **(D)** Confusion matrix derived from the test set.

Therefore, based on comprehensive evaluation, XGBoost was selected as the final model. Further evaluation using the Kolmogorov–Smirnov curve demonstrated good discriminative ability of the model, with a KS statistic of 0.569 at a threshold of 0.010. Learning curve analysis indicated that model performance gradually stabilized as the training sample size increased, with no evident signs of overfitting or underfitting. Importantly, confusion matrix analysis was carefully re-examined to ensure correct interpretation in the context of class imbalance. In the training cohort, the model correctly identified 1,341 true negatives (non-PE cases) and 20 true positives (PE cases). In the testing cohort, 565 true negatives and 2 true positives were correctly classified, reflecting the inherently low incidence of postoperative pulmonary embolism in the dataset (1.38%) and the corresponding rarity of positive events. These results indicate that the high overall classification accuracy observed in both cohorts is primarily driven by correct identification of the majority (non-PE) class. Therefore, performance interpretation was further complemented by class-specific metrics, particularly sensitivity for PE detection, to provide a more clinically meaningful assessment of model performance under imbalanced outcome conditions (Figures 5A–D).

### Rigorous external validation to assess generalizability across independent cohorts

**Figure 6:**
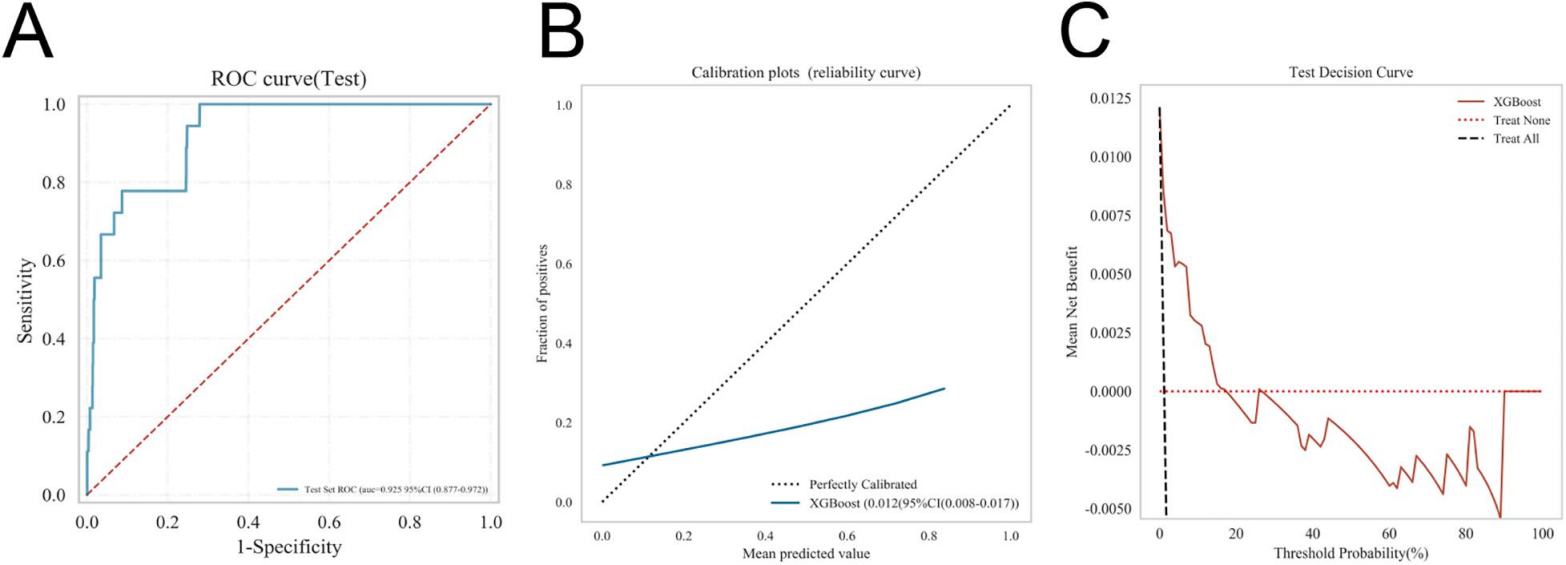
External validation of the XGBoost predictive model. The generalizability and cross-cohort applicability of the XGBoost model were evaluated utilizing an independent, geographically distinct external dataset. **(A)** ROC curve demonstrating the discriminative performance of the model in the external validation cohort, with the area under the curve (AUC) providing a quantitative summary of predictive accuracy. **(B)** Calibration curve assessing the alignment between predicted probabilities and observed outcome proportions, wherein the calibration slope and intercept serve as indices of model reliability. **(C)** Decision curve analysis (DCA) quantifying the net clinical benefit conferred by the model across a spectrum of threshold probabilities in the external validation setting.

In the external validation cohort, the model demonstrated excellent discriminative performance, with an AUC of 0.925 (95% CI: 0.877–0.972). Calibration analysis showed good agreement between predicted and observed outcomes, with a Brier score of 0.012 (95% CI: 0.008–0.017), indicating satisfactory calibration ability. Furthermore, DCA demonstrated favorable net clinical benefit across a wide range of threshold probabilities, suggesting that the model has strong potential for clinical application (Figures 6A–C).

### SHAP-Based interpretability to provide clinically meaningful explanations of individual-level and global feature contributions

**Figure 7:**
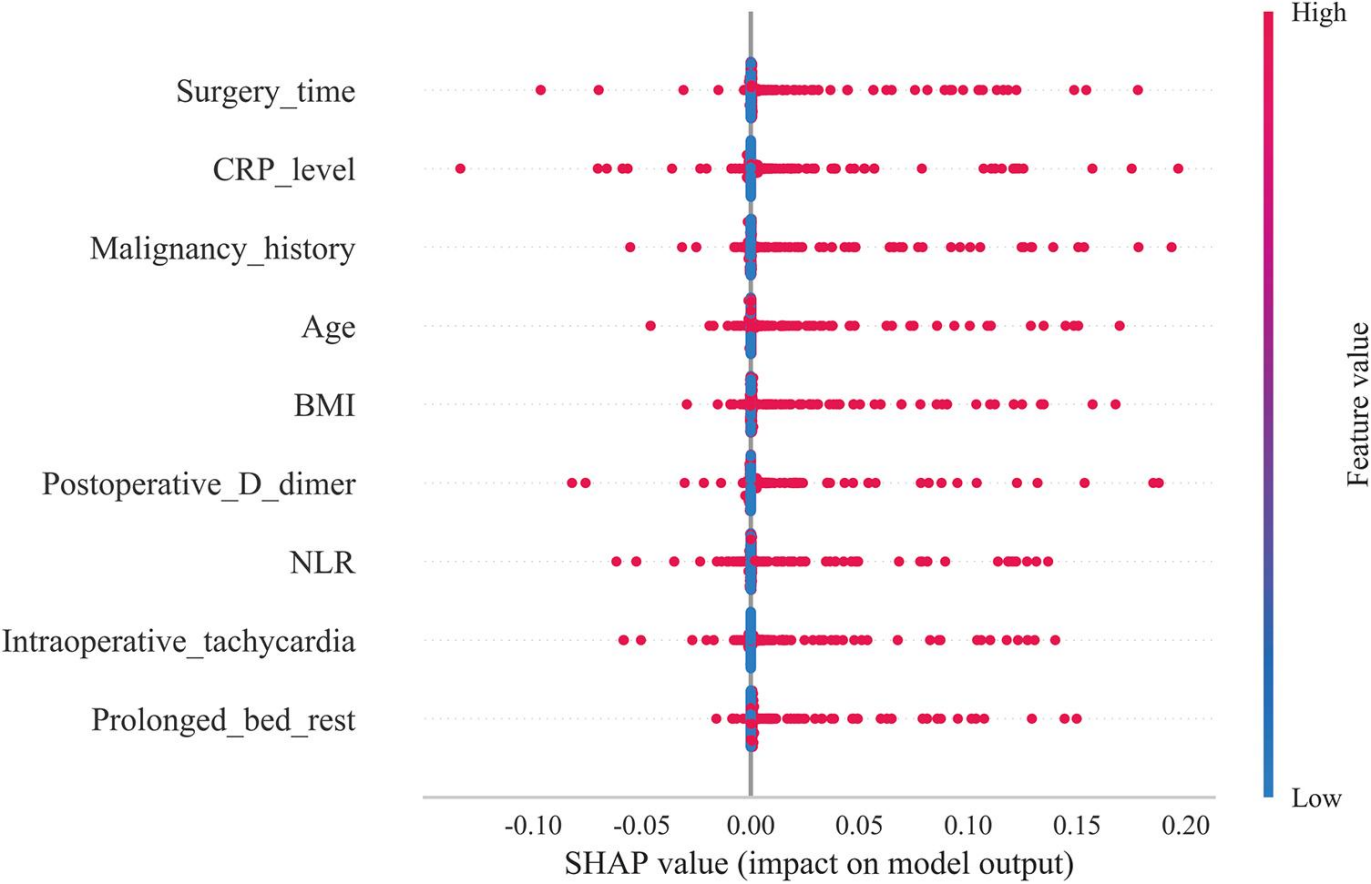
Global interpretability of the XGBoost model as revealed by SHapley additive exPlanations (SHAP) analysis. To enhance the transparency of the XGBoost framework, SHAP analysis was conducted. The SHAP summary plot furnishes a comprehensive overview of feature importance, delineating the aggregate contribution of each predictor variable to the model's output. The color gradient encodes the magnitude of the feature value, whereas the position along the horizontal axis denotes the directionality and magnitude of the impact on the predicted outcome. This visualization facilitates a population-level understanding of the principal determinants governing postoperative pulmonary embolism risk.

To enhance the interpretability of the predictive model, SHAP analysis was performed to quantify the contribution of each variable to postoperative pulmonary embolism risk. The SHAP summary plot revealed that the most influential predictors, ranked in descending order of importance, were surgery time, elevated CRP, malignancy history, advanced age, increased BMI, elevated postoperative D-dimer, increased NLR, intraoperative tachycardia, and prolonged postoperative bed rest (Figure 7). These findings highlight the combined contribution of surgical burden, inflammatory response, coagulation activation, and baseline patient characteristics in driving PE risk.

**Figure 8:**
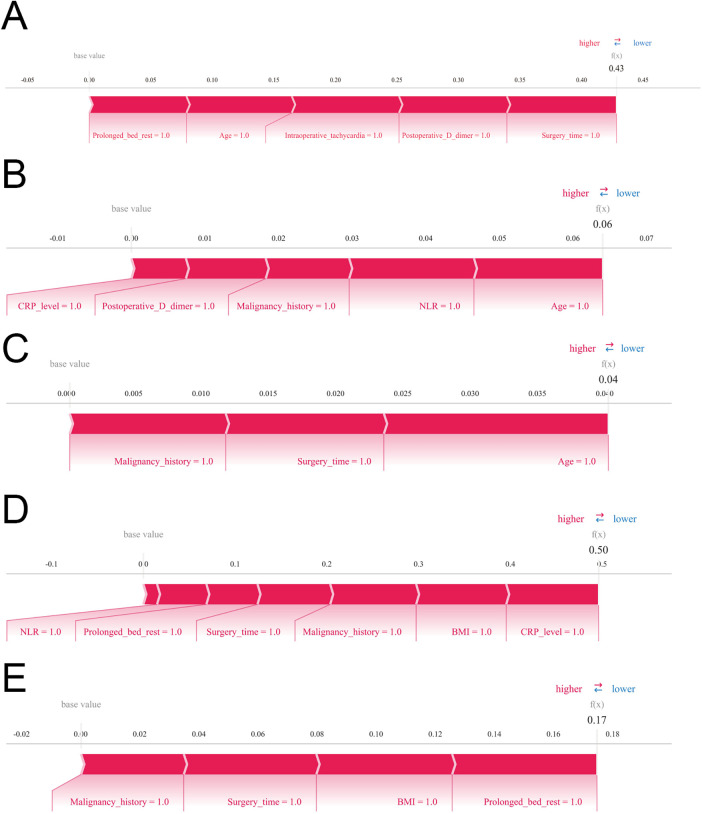
Individual-level interpretability of the XGBoost model illustrated via SHAP force plots. **(A–E)** Representative examples derived from five individual patient cases. To enable personalized risk deconvolution, SHAP force plots were generated to explicate the model's decision logic at the granularity of the single patient, thereby delineating the additive contribution of specific clinical features to the final individualized risk estimate.

Furthermore, SHAP decision plots were used to provide individualized explanations for five representative patients with postoperative PE, illustrating the key factors contributing to model predictions at the individual level (Figures 8A–E).

Patient 1 had a predicted probability of 0.43, corresponding to a moderate-risk category. The SHAP decision plot indicated that prolonged postoperative bed rest, advanced age, intraoperative tachycardia, elevated postoperative D-dimer, and prolonged surgery time were the primary contributors driving the prediction toward increased risk.

Patient 2 showed a relatively low predicted probability (0.06). Despite this, elevated postoperative D-dimer, increased NLR and CRP levels, malignancy history, and older age contributed positively to risk, although their combined effect was insufficient to shift the overall prediction into a high-risk category.

Patient 3 had a predicted probability of 0.04, indicating low risk. The prediction was mainly influenced by malignancy history, advanced age, and prolonged surgery time; however, the overall contribution of these factors remained limited, resulting in a low-risk classification.

Patient 4 demonstrated a higher predicted probability (0.50), corresponding to a moderate-to-high risk profile. The key driving factors included elevated postoperative inflammatory markers (NLR and CRP), malignancy history, prolonged bed rest, prolonged surgery time, and increased BMI.

Patient 5 had a predicted probability of 0.17. The model indicated that malignancy history, prolonged surgery time, elevated BMI, and prolonged postoperative bed rest were the main contributors to the increased risk.

**Figure 9:**
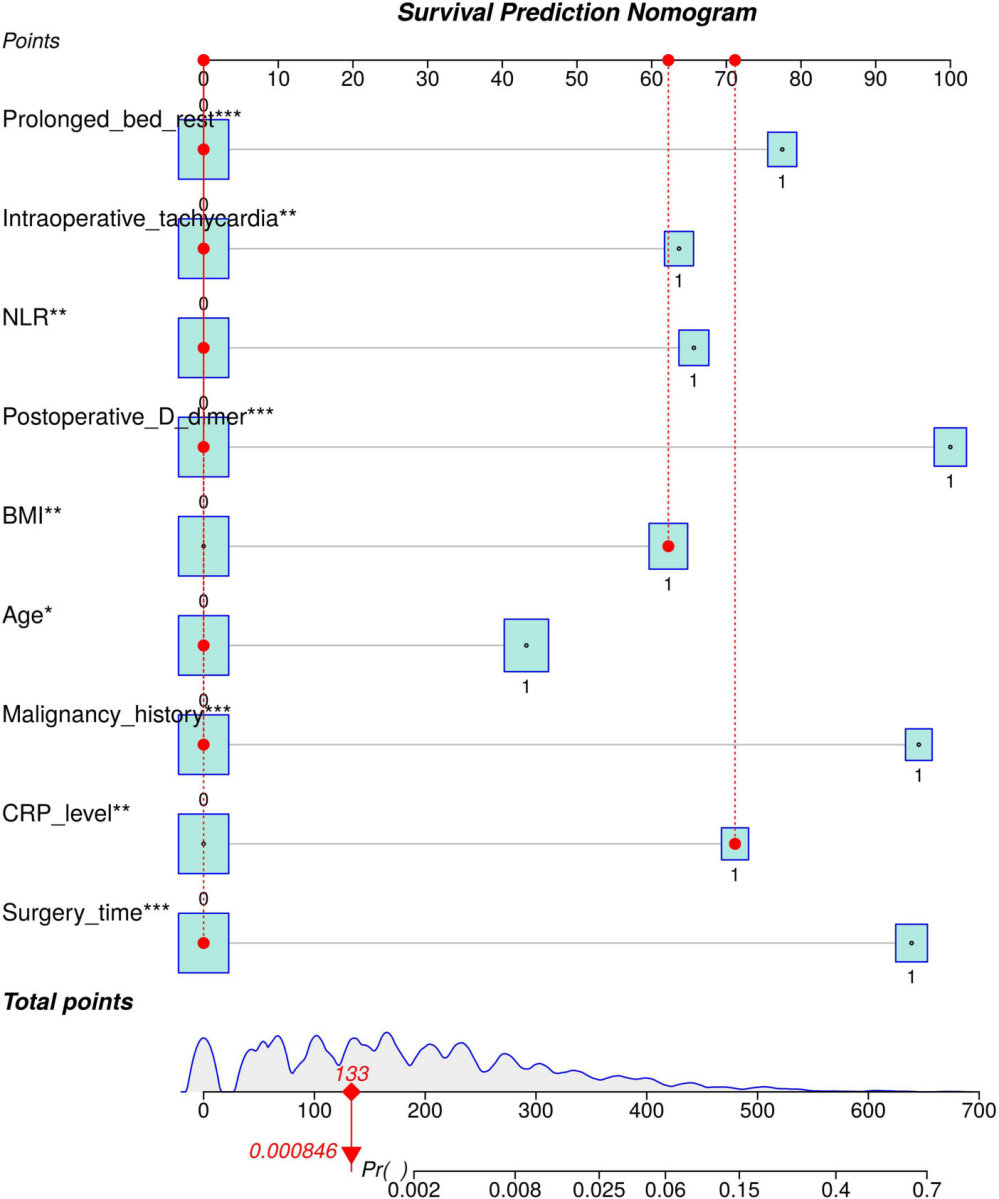
Nomogram for predicting postoperative pulmonary embolism risk.

In this study, a nomogram was constructed integrating important predictor variables including age, body mass index (BMI), malignancy history, prolonged bed rest, surgery time, intraoperative tachycardia, C-reactive protein (CRP), neutrophil-to-lymphocyte ratio (NLR), and postoperative D-dimer, aiming to visually assess the risk of postoperative pulmonary embolism. Figure 9 presents an interactive nomogram, in which the example patient achieved a total score of 133 points, corresponding to a predicted probability of pulmonary embolism of 0.0846%, thereby providing a rapid and easily interpretable tool for clinical risk assessment.

## Discussion

In the present study, both a conventional Logistic regression–based nomogram and multiple machine learning models were developed to evaluate postoperative pulmonary embolism risk, providing an opportunity to compare traditional scoring systems with modern data-driven approaches in perioperative risk prediction. Conventional regression-based scoring systems, such as nomograms derived from Logistic regression, offer several important advantages. First, they are inherently interpretable, as each variable is assigned a fixed coefficient that directly reflects its average effect on the outcome within the study population. This facilitates straightforward clinical understanding and enables bedside calculation of individualized risk scores without computational tools. Second, such models are generally more transparent and easier to validate across institutions, making them more acceptable in guideline development and clinical decision-making frameworks. Third, their additive structure allows for simple translation into point-based scoring systems, which enhances usability in routine clinical practice. However, traditional scoring systems also have notable limitations. They assume linearity and additivity among predictors, which may not adequately capture the complex, nonlinear, and interactive relationships that exist among perioperative inflammation, coagulation activation, and hemodynamic instability. In addition, regression-based models may be less sensitive to subtle high-order interactions between variables, potentially limiting their predictive performance in heterogeneous clinical populations. In contrast, machine learning models, particularly ensemble methods such as XGBoost, demonstrated superior discriminative ability in the present study. Their primary strength lies in the capacity to model complex nonlinear relationships and high-dimensional interactions among clinical variables without pre-specifying functional forms. This is particularly relevant in postoperative pulmonary embolism, where risk is driven by the interplay of multiple dynamic processes, including inflammatory response, thrombogenesis, surgical stress, and patient-specific baseline characteristics. Furthermore, the incorporation of SHAP-based interpretability enhances the transparency of machine learning models by enabling both global feature ranking and individualized risk attribution, thereby partially addressing the traditional "black-box" limitation. Nevertheless, machine learning models are not without limitations. First, their interpretability, although improved through SHAP, remains less intuitive than coefficient-based scoring systems for direct bedside use. Therefore, in this study, a complementary approach combining the Logistic regression–based nomogram and machine learning models was adopted. This approach not only provides a simplified and clinically intuitive bedside risk assessment tool using the nomogram but also leverages the superior predictive performance of machine learning models to capture complex perioperative interactions that may be overlooked by linear models. The combined use of both methods enhances the interpretability and predictive robustness of the proposed risk stratification framework, thereby improving its potential clinical utility in the perioperative management of patients at risk for pulmonary embolism.

In the present study, five machine learning algorithms—namely KNN, SVM, RF, MLP, and XGBoost—were selected to construct and comparatively evaluate predictive models for postoperative pulmonary embolism. The rationale for selecting this ensemble of algorithms was to encompass a broad spectrum of modeling paradigms, including distance-based learning (KNN), kernel-based classification (SVM), ensemble tree-based methods (RF and XGBoost), and neural network-based architectures (MLP). This multi-algorithm strategy enables a comprehensive appraisal of model performance under divergent assumptions regarding data structure, feature interactions, and non-linearity, thereby enhancing the robustness of model selection (15, 24, 25). Among the evaluated models, XGBoost demonstrated superior overall performance with respect to discrimination, calibration, and clinical utility. The preeminence of XGBoost may be attributable to its gradient boosting architecture, which sequentially optimizes weak learners to minimize residual error, thereby effectively capturing complex non-linear relationships and high-order feature interactions inherent in perioperative clinical data. Furthermore, its built-in regularization mechanisms mitigate the risk of overfitting, a consideration of particular salience in clinical datasets characterized by class imbalance and heterogeneous feature distributions. Compared with alternative algorithms, XGBoost afforded a more favorable balance between predictive accuracy and generalizability, rendering it especially suitable for clinical risk prediction tasks (26).

To further enhance the clinical interpretability of the model, SHAP analysis was conducted to quantify the contribution of individual variables to model predictions. The analysis revealed that operative duration, postoperative inflammatory markers (including CRP and NLR), history of malignancy, advanced age, elevated BMI, elevated postoperative D-dimer concentration, and intraoperative tachycardia constituted the most influential predictors of postoperative pulmonary embolism. The predictive salience of the key variables identified by the model can be systematically mechanistically explicated within the conceptual framework of Virchow's triad, wherein each clinical parameter maps onto distinct dimensions of the core pathophysiological pathways governing the initiation and progression of pulmonary embolism, reflecting the synergistic interplay among endothelial injury, hemodynamic alterations, and hypercoagulability.

Within the Virchow triad framework, prolonged operative duration can be conceptualized as a cumulative index of endothelial injury. Its impact extends beyond direct macroscopic vascular trauma to encompass microvascular endothelial dysfunction induced by sustained intraoperative retraction, thermal diffusion from electrocautery, and ischemia–reperfusion processes (27, 28). Emerging evidence (29–32) indicates that the endothelial glycocalyx layer plays a pivotal role in this cascade. The glycocalyx, a dynamic barrier structure composed of proteoglycans and glycosaminoglycans, serves a critical function in maintaining hemodynamic shear stress sensing and anticoagulant homeostasis. Surgical trauma and the attendant inflammatory response can precipitate glycocalyx degradation, accompanied by elevated circulating levels of its constituent components, such as syndecan-1 and heparan sulfate, a phenomenon that has been empirically associated with the extent of surgical insult and clinical prognosis (30, 33). Following glycocalyx disruption, the subendothelial matrix becomes exposed, enabling direct contact between type I and type III collagen and circulating blood elements. This exposure facilitates initial platelet adhesion via the interaction between the glycoprotein Ib-IX-V complex and von Willebrand factor (vWF). Concurrently, subendothelial cells and activated fibroblasts express tissue factor, which forms a complex with coagulation factor VIIa, thereby initiating the extrinsic coagulation cascade and triggering thrombin generation (34, 35). Accordingly, operative duration functions within the model not merely as a temporal variable but as an indirect quantitative proxy for the cumulative burden of endothelial injury and the extent of tissue factor exposure.

Conversely, prolonged postoperative immobilization promotes venous stasis through inhibition of the calf muscle pump function. At the molecular and biomechanical level, however, the core mechanism is localized to the venous valvular sinus region. Under conditions of physiological laminar shear stress, endothelial cells maintain an anti-inflammatory and anticoagulant phenotype through activation of Krüppel-like factor 2 (KLF2). Postoperative immobilization engenders a low-shear or oscillatory flow state, thereby suppressing the MEK5/ERK5-KLF2 signaling axis and inducing a phenotypic shift of endothelial cells toward a prothrombotic state. The consequences of this shift include downregulation of thrombomodulin expression, increased release of vWF, and upregulation of plasminogen activator inhibitor-1 (PAI-1), collectively establishing a localized microenvironment characterized by hypercoagulability and hypofibrinolysis (36, 37).

Our findings further corroborated that a history of malignancy confers a markedly elevated risk of postoperative pulmonary embolism, a phenomenon reflective of tumor-associated systemic hypercoagulability. One salient mechanism underlying cancer-associated thrombosis involves the release of circulating tumor-derived microparticles. These microparticles frequently express tissue factor and expose phosphatidylserine on their surface, thereby serving as a scaffold for the assembly of coagulation complexes and promoting the coagulation cascade at vascular sites remote from the primary tumor (38, 39). Additionally, certain adenocarcinomas, particularly mucinous subtypes, secrete mucins rich in glycosylated structures. Through interactions with P-selectin and L-selectin, these molecules facilitate platelet–leukocyte aggregation, thereby potentiating thrombotic propensity. It warrants clarification that while mucins substantially amplify the coagulation response, they do not directly cleave prothrombin; rather, they indirectly augment thrombin generation by promoting the assembly of coagulation complexes and enhancing cellular interactions (40). Consequently, patients with malignant neoplasms frequently exist in a state of persistent subclinical hypercoagulability, with surgical trauma acting as the critical precipitating trigger for overt clinical thrombotic events.

Nevertheless, the mere presence of the three constituent elements of Virchow's triad is insufficient to invariably precipitate clinical thrombotic events. The model further elucidated that the postoperative acute inflammatory response serves as a crucial triggering factor driving the transition from a hypercoagulable state to clinically manifest pulmonary embolism. The present study identified postoperative inflammatory indices (NLR and CRP) and D-dimer dynamics as possessing substantial predictive value, the mechanisms of which can be explicated through the dual lenses of immunothrombosis and fibrinolytic suppression.

First, an elevated NLR signifies an intensified neutrophil-predominant inflammatory response and is intimately associated with the formation of neutrophil extracellular traps (NETs) (41). NETs, composed of a decondensed DNA scaffold studded with histones and granular proteins, exert a dual function as both a physical scaffold and a biochemical amplifier in the process of thrombus formation. At the physical level, the reticular architecture of NETs markedly enhances the adhesion of platelets, erythrocytes, and vWF, thereby promoting thrombus stabilization and propagation. At the biochemical level, neutrophil elastase tethered to NETs can inactivate tissue factor pathway inhibitor (TFPI), thereby attenuating the endogenous negative regulation of the extrinsic coagulation pathway, whereas histones can activate coagulation factor XII, thereby triggering the intrinsic coagulation cascade (42–44). Hence, NETosis establishes a critical nexus between inflammation and coagulation, enabling the rapid transduction of a localized inflammatory response into systemic procoagulant signaling.

Second, elevated CRP levels may further amplify perioperative thrombotic susceptibility through complex interactions involving inflammatory activation, coagulation cascade enhancement, endothelial dysfunction, and impaired fibrinolysis. Importantly, the contribution of CRP identified by the SHAP model should not be interpreted as a fixed or isolated causal effect; rather, its impact is context-dependent and varies according to the overall perioperative thrombo-inflammatory milieu of individual patients. In the present study, SHAP analysis demonstrated that the predictive contribution of CRP differed across patients with distinct combinations of surgical burden, coagulation status, hemodynamic instability, malignancy background, and postoperative immobilization. This observation is clinically plausible, as postoperative pulmonary embolism is fundamentally a multifactorial process driven by the dynamic interplay among inflammation, hypercoagulability, venous stasis, and endothelial injury. From a mechanistic perspective, CRP elevation commonly reflects bacterial-associated inflammation or severe systemic inflammatory activation, with one of the central molecular pathways involving activation of the lipopolysaccharide (LPS)/Toll-like receptor 4 (TLR4) signaling axis. Activation of this pathway can upregulate tissue factor transcription and membrane surface expression within the monocyte–macrophage system while simultaneously promoting the release of circulating procoagulant microparticles, thereby enhancing initiation of the extrinsic coagulation pathway. Concurrently, LPS/TLR4 signaling may induce increased endothelial expression of plasminogen activator inhibitor-1 (PAI-1), suppress tissue-type plasminogen activator-mediated plasmin generation, and impair endogenous fibrinolytic activity directed against newly formed thrombi (45, 46). Although CRP is conventionally regarded as a non-specific inflammatory biomarker in clinical practice, accumulating evidence suggests that CRP itself may actively participate in thrombo-inflammatory regulation. Experimental studies have demonstrated that CRP can induce tissue factor expression through Fc*γ* receptor-mediated activation of monocytes and further promote endothelial dysfunction and PAI-1 upregulation, thereby reinforcing the pathological imbalance between hypercoagulability and hypofibrinolysis (47, 48). However, the extent to which CRP contributes to postoperative PE risk likely depends on the concomitant presence of additional perioperative stressors, including prolonged operative duration, elevated postoperative D-dimer, malignancy-associated prothrombotic states, intraoperative tachycardia, advanced age, and prolonged immobilization. Therefore, the individualized SHAP decision plots presented in this study should not be interpreted as demonstrating isolated biomarker effects, but rather as visual representations of how multiple interacting perioperative factors collectively shift patient-specific thromboembolic risk. In this context, postoperative inflammation may function not merely as a passive epiphenomenon, but as a dynamic pathological component integrated within a broader perioperative thrombo-inflammatory network that cooperatively drives progression toward postoperative pulmonary embolism.

Notably, whereas preoperative D-dimer concentration was included as a candidate variable in the present study, its predictive utility for postoperative pulmonary embolism did not attain statistical significance. This finding stands in contrast to the pronounced predictive value observed for postoperative D-dimer and suggests that D-dimer carries fundamentally distinct pathophysiological connotations at different temporal phases of the perioperative period. Preoperative D-dimer predominantly reflects the baseline fibrin turnover status of the patient; its elevation may stem from age-related augmentation of coagulation activity, tumor-associated hypercoagulability, or chronic low-grade inflammation (49). However, this static metric largely mirrors the prior or long-term equilibrium of the coagulation–fibrinolysis axis and lacks sensitivity to acute perioperative pathophysiological perturbations, thereby rendering it suboptimal for predicting *de novo* surgically precipitated thrombotic events (50, 51). In contradistinction, against the backdrop of the inflammation-driven procoagulant milieu delineated above, the elevation of postoperative D-dimer must be interpreted comprehensively through the dual dimensions of increased generation and impaired clearance, essentially reflecting a dynamic disequilibrium among coagulation activation, fibrinolytic response, and metabolic elimination (52, 53). On the one hand, fibrin formation and degradation during postoperative wound healing can result in a physiological elevation of D-dimer. On the other hand, persistent or aberrant elevation is more likely indicative of secondary fibrinolytic activation consequent to occult venous thrombus formation, thereby mirroring the pathological process of ongoing thrombogenesis coupled with secondary fibrinolysis. More importantly, the inflammatory response exerts an amplifying effect within this dynamic. Systemic inflammation can compromise the function of the hepatic mononuclear phagocyte system, thereby diminishing the clearance capacity for fibrin degradation products, prolonging the plasma half-life of D-dimer, and amplifying its measured concentration. Concurrently, inflammatory mediators further perturb its metabolic processing through modulation of the hepatic acute-phase response and hemodynamic status, conferring upon D-dimer a kinetic profile characterized by the coexistence of enhanced generation and restricted clearance. Consequently, within the framework of the present study, D-dimer should not be construed as a simplistic unidimensional marker of coagulation activation; rather, it is more aptly conceptualized as an integrated biological readout of the continuum encompassing inflammation-driven coagulation generation, fibrinolytic response, and clearance impairment. Its synergistic elevation in conjunction with postoperative inflammatory indices signals that the organism has entered a state of disequilibrium marked by hypercoagulability, hypofibrinolysis, and compromised clearance—a state that may herald the critical window of transition from subclinical thrombosis to clinically overt pulmonary embolism. From a temporal perspective, the divergence between preoperative and postoperative D-dimer essentially delineates the distinction between baseline susceptibility and event-triggering status: the former represents the background level of long-term individual thrombotic propensity, whereas the latter integrates the immediate effects of surgical trauma, inflammatory activation, and endothelial injury. Accordingly, compared with single-time-point baseline measurements, the dynamic trajectory of postoperative D-dimer is better positioned to capture pivotal inflection points in the thrombotic process, thereby manifesting superior predictive performance.

Furthermore, the SHAP-based individualized analysis conducted in this study revealed that age and body mass index, as fundamental host-specific characteristics, exert significant modulating effects on susceptibility to postoperative pulmonary embolism. The underlying essence resides in the capacity of these two factors to induce long-term remodeling effects on vascular endothelial homeostasis and the coagulation–inflammation network. In the context of advancing age, vascular endothelial function undergoes progressive decline, a process manifest not only as structural alterations but also as functional disequilibrium at the molecular level. One of the most consequential changes is the diminished bioavailability of endothelium-derived nitric oxide, attributable to both downregulated expression of endothelial nitric oxide synthase and enhanced nitric oxide inactivation driven by oxidative stress (54–56). Nitric oxide, as a pivotal endogenous antithrombotic mediator, inhibits platelet aggregation, attenuates leukocyte adhesion, and sustains vasodilation; its depletion directly compromises the anticoagulant and anti-inflammatory capacity of the vasculature. Concomitantly, circulating levels of vWF and coagulation factor VIII exhibit progressive elevation with advancing age, propelling the plasma milieu toward a relative hypercoagulable state (57–59). This dual alteration—characterized by diminished endothelial protection coupled with enhanced coagulation drive—renders elderly patients more susceptible to surpassing the physiological threshold for thrombus formation when confronted with surgical trauma. Conversely, the obese state, as represented by elevated BMI, further amplifies this risk through mechanisms involving chronic low-grade inflammation and metabolic dysregulation. Adipose tissue is now unequivocally recognized as an active endocrine and immune organ, with visceral adipose tissue in particular capable of constitutively secreting a repertoire of proinflammatory and procoagulant mediators, including tumor necrosis factor-*α*, interleukin-6, and PAI-1. These factors not only directly promote coagulation but also suppress fibrinolytic activity, thereby impeding thrombus resolution (60, 61). Moreover, leptin secreted by adipocytes can upregulate tissue factor expression in monocytes and endothelial cells via the JAK/STAT signaling pathway, further sensitizing the coagulation cascade. It merits emphasis that a notable synergistic effect exists between obesity-related chronic low-grade inflammation and the postoperative acute inflammatory response (62, 63). In obese patients, the basal inflammatory milieu already resides in a pre-activated state, characterized by monocyte priming and mild endothelial dysfunction. When surgical trauma elicits an acute inflammatory response, this primed state enables the coagulation system to undergo rapid amplification and activation at a lower threshold, engendering a pathological process akin to a priming effect. Consequently, compared with their non-obese counterparts, obese individuals are predisposed to a more rapid transition from subclinical hypercoagulability to clinically manifest thrombotic events in the early postoperative period.

In the present study, intraoperative tachycardia was further identified as a novel and independent perioperative risk marker for postoperative pulmonary embolism. This finding underscores that intraoperative tachycardia should not be construed merely as a transient hemodynamic perturbation but rather as an integrative physiological phenotype reflecting concurrent alterations in autonomic activation, circulatory stress, and incipient inflammatory signaling during the intraoperative period. From a hemodynamic standpoint, intraoperative tachycardia is frequently associated with sympathetic nervous system activation triggered by hypovolemia, nociceptive stimulation, hypoxemia, or the systemic inflammatory response (64, 65). Sustained elevation of heart rate abbreviates diastolic filling time, thereby attenuating effective venous return and promoting venous pooling, particularly within the deep veins of the lower extremities. This phenomenon of “tachycardia-associated venous stasis” may be further compounded under general anesthesia and specific intraoperative positioning, collectively contributing to blood flow stagnation—a cardinal component of Virchow's triad. At the microcirculatory level, hemodynamic redistribution induced by tachycardia may engender regional zones of diminished shear stress despite preserved or even augmented global cardiac output. Such low-shear microenvironments, notably within venous valve sinuses, are recognized to promote endothelial phenotypic switching toward a prothrombotic state. Mechanistically, reduced shear stress suppresses Krüppel-like factor 2 (KLF2) signaling, culminating in downregulation of thrombomodulin and endothelial protein C receptor expression, while concomitantly potentiating antifibrinolytic pathways, thereby facilitating local thrombus initiation and propagation. Moreover, intraoperative tachycardia may directly contribute to endothelial dysfunction via catecholamine-mediated oxidative stress. Sustained sympathetic activation diminishes nitric oxide bioavailability, thereby compromising endothelial anti-inflammatory and antithrombotic properties. Furthermore, tachycardia frequently coexists with fluctuating intravascular volume status and intermittent hypotensive episodes, engendering dynamic shear stress variability that further augments endothelial activation and tissue factor expression. Importantly, intraoperative tachycardia may also serve as an early clinical surrogate of systemic inflammatory activation. Accumulating evidence indicates a close association between elevated heart rate and circulating inflammatory mediators, including interleukin-6 and tumor necrosis factor-*α* (66–69). In this context, tachycardia may not merely reflect hemodynamic stress but may also portend the early initiation of immunothrombotic cascades, thereby bridging intraoperative physiological perturbation with subsequent postoperative elevations in NLR, CRP, and procalcitonin. Collectively, these observations suggest that intraoperative tachycardia ought to be interpreted as a dynamic integrative marker linking autonomic dysregulation, venous stasis, endothelial dysfunction, and early immunothrombosis, thus furnishing a novel mechanistic perspective for perioperative risk stratification of pulmonary embolism.

Importantly, while the present model demonstrated strong discriminative performance as reflected by AUC-based evaluation, it is essential to recognize that high AUC values do not necessarily translate into high absolute detection rates for postoperative pulmonary embolism in clinical practice. This distinction is particularly critical in the context of rare-event outcomes, where even well-performing models may yield a limited number of true positive cases due to the inherently low prevalence of the condition. In this study, although the XGBoost model achieved robust performance across multiple evaluation metrics, including discrimination, calibration, and decision curve analysis, the absolute number of correctly identified PE cases remained limited. This reflects the low incidence of the outcome rather than a limitation of the model itself. Therefore, model evaluation should extend beyond aggregate metrics and incorporate class-specific performance, particularly sensitivity for PE detection, when assessing real-world clinical utility. Accordingly, the clinical role of the proposed model should be clearly defined. It should be regarded primarily as a risk stratification and early warning tool rather than a definitive diagnostic instrument. Its principal value lies in identifying patients at elevated risk of postoperative pulmonary embolism who may benefit from closer surveillance, intensified perioperative monitoring, or early preventive interventions, rather than directly confirming or excluding the diagnosis of pulmonary embolism. This interpretation is consistent with the intended application of machine learning–based perioperative prediction models, which are designed to support clinical decision-making by prioritizing high-risk individuals within large surgical populations, rather than replacing established diagnostic modalities such as imaging-based confirmation.

This study has several limitations. First, its retrospective design introduces unavoidable risks of selection and information bias, despite standardized data collection procedures. Second, the external validation cohort was derived from a relatively confined geographic and healthcare system context, which may limit generalizability to more heterogeneous populations. Third, several potentially relevant biomarkers related to thrombosis, endothelial dysfunction, and inflammatory activation were not available in the dataset, limiting the comprehensiveness of feature representation. Importantly, the number of postoperative pulmonary embolism events was relatively small (*n* = 48, 1.38%), representing an inherent limitation of rare-event predictive modeling. Although stratified cross-validation and independent external validation were employed to improve robustness, such a limited event count may still affect model stability and increase uncertainty in parameter estimation, particularly for complex machine learning approaches. In addition, differences in case mix, perioperative management strategies, and institutional practice patterns may contribute to performance variability across datasets. Model interpretability was derived from SHAP-based *post hoc* analyses, which provide valuable insights into feature contributions at both global and individual levels; however, these explanations remain associative and should not be interpreted as evidence of causality. Finally, perioperative management strategies, including thromboprophylaxis and anticoagulation protocols, were not fully standardized across centers, introducing potential residual confounding. Future prospective multicenter studies with larger sample sizes, higher event counts, and more standardized perioperative management protocols are warranted to further validate, calibrate, and refine the proposed model, and to enhance its robustness and clinical applicability across diverse healthcare settings.

## Conclusion

In this multicenter investigation, we developed and externally validated a machine learning-based predictive model for postoperative pulmonary embolism. The model exhibited favorable discrimination, calibration, and clinical utility across both internal and external validation cohorts, indicative of robust generalizability. Among the five algorithms evaluated, XGBoost demonstrated superior overall performance. Multiple complementary evaluation methodologies—including receiver operating characteristic analysis, calibration curve plotting, and decision curve analysis—corroborated the model's robustness and clinical applicability. SHAP analysis delineated key predictive determinants, encompassing inflammatory markers (CRP, NLR, postoperative D-dimer), surgical parameters (operative duration, intraoperative tachycardia), and host baseline characteristics (age, BMI, history of malignancy, and prolonged immobilization). These findings collectively underscore the synergistic contribution of postoperative inflammation and surgical stress to the pathogenesis of pulmonary embolism in the perioperative setting.

## Data Availability

The original contributions presented in the study are included in the article/Supplementary Material, further inquiries can be directed to the corresponding author.
